# Analyzing the transient response dynamics of long-term depression in the mouse auditory cortex *in vitro* through multielectrode-array-based spatiotemporal recordings

**DOI:** 10.3389/fnins.2024.1448365

**Published:** 2024-09-12

**Authors:** Ryo Furukawa, Kouta Kume, Takashi Tateno

**Affiliations:** ^1^Graduate School of Information Science and Technology, Hokkaido University, Sapporo, Japan; ^2^Faculty of Information Science and Technology, Hokkaido University, Sapporo, Japan

**Keywords:** auditory cortex, electric stimulation, multielectrode array, high-frequency stimulation, local cortical network, long-term depression, spatiotemporal neural plasticity

## Abstract

In the auditory cortex, synaptic plasticity, including long-term potentiation (LTP) and long-term depression (LTD), plays crucial roles in information processing and adaptation to the auditory environment. Previous rodent studies have shown lifelong cortical map plasticity, even beyond the critical period of development. While thalamocortical synapses exhibit LTD during the critical period, little is known about LTD in the cortico-cortical connections of the adult mouse auditory cortex. Here, we investigated the transient response dynamics of LTD in layers 2–5 of the mouse auditory cortex following tetanic stimulation (TS) to layer 4. To characterize LTD properties, we developed a recording protocol to monitor activity levels at multiple sites, including those more than 0.45 mm from the TS site. This allowed us to distinguish LTD-induced reductions in neural excitability from other types, including neural activity depletion. Our findings revealed that LTD induced in layer 4 persisted for over 40-min post-TS, indicating robust cortico-cortical LTD. Using electrophysiological data and a modified synaptic model, we identified key receptors involved in synaptic plasticity and their effects on response dynamics, proposing a method for studying LTD in the mature mouse auditory cortex. Particularly, by employing a simple dynamical model, we analyzed and discussed the involvement of key receptors during the transient period of LTD. This study expands our understanding of synaptic plasticity in the mature mouse auditory cortex beyond the critical period, potentially informing future treatments for hearing disorders.

## 1 Introduction

Cortical maps in the auditory cortex of mammalians are plastic and activity-dependently changing in response to auditory experience. Synaptic plasticity in the auditory cortex is believed to underlie the mechanisms for the adaptive feature changes of auditory perception. In the rodent auditory cortex, tonotopic representation of acoustic signals is formed during development and is changing in an experience-dependent manner (Buonomano and Merzenich, 1998; Weinberger, 2004), suggesting the involvement of synaptic plasticity in this adaptive process. In rodents, including mice and rats, previous studies revealed that cortical map plasticity can be induced in the auditory cortex throughout the lifespan during and even after the critical period (Zhang et al., 2001; de Villers-Sidani et al., 2007; Insanally et al., 2009; Dorrn et al., 2010). However, long-term potentiation (LTP) and long-term depression (LTD), which are likely to be the mechanisms of synaptic plasticity, can be induced at thalamocortical synapses in brain slices by electrical stimulation of thalamic projections, but only during an early critical period that corresponds to the first several postnatal days in rodents (Crair and Malenka, 1995; Barth and Malenka, 2001). In addition, several studies have reported that both LTP and LTD at thalamocortical synapses cannot be induced only in brain slices taken from rodents older than 2–3 weeks (Kirkwood et al., 1995; Isaac et al., 1997; Feldman and Knudsen, 1998). In contrast, Blundon et al. found that in brain slices taken from mature mice aged 7–8 weeks, LTD and LTP are not lost at thalamocortical synapses after the critical period but rather become gated, persisting with the activation of presynaptic M1 cholinergic receptors (M1Rs) on thalamic inputs in the rodent auditory cortex (Blundon et al., 2011; Blundon and Zakharenko, 2013; Chun et al., 2013). Therefore, knowledge on synaptic plasticity at thalamocortical synapses in the rodent auditory cortex has been accumulating to some extent. However, little is known about activity-dependent synaptic plasticity of cortico-cortical connections in the matured rodent auditory cortex beyond the auditory critical period, particularly over postnatal week 7. Furthermore, the direct modification of synaptic plasticity induced by electrical stimulation of the auditory cortex is a future possibility to be applied to the medical treatment of hearing disorders such as hearing impairment, presbycusis, and tinnitus. However, with regard to the application of high-frequency electric stimulation to the auditory cortex, the detailed properties of the plastic changes in cortico-cortical synapses have not been examined.

As a major form of long-term synaptic plasticity, the present study focuses on and examines *in vitro* LTP/LTD at matured cortico-cortical synapses of the auditory cortex obtained from young adult mice (postnatal week 7 and more). Particularly, we applied high-frequency (tetanic) stimulation to layer 4 in the mouse auditory cortex slices to characterize the plastic changes of neural activity before and after the tetanic stimulation (TS). In the rodent hippocampal slices, the TS is usually used to induce LTP at the mossy fiber synapses onto CA3 pyramidal neurons (Bliss and Lomo, 1973; Bliss and Collingridge, 1993; Davis et al., 1997) and LTP at Schaffer collateral synapses onto CA1 pyramidal neurons (Zalutsky and Nicoll, 1990). However, such type of long-term synaptic plasticity has not been extensively examined in mouse cortical slices. Although several research groups reported the results of long-term synaptic plasticity in rat cortical slices (Kudoh and Shibuki, 1994; Murakami et al., 2004; Watanabe et al., 2007), direct thalamocortical projections from the medial geniculate body (MGB) were eliminated, and cortico-cortical connections were partly retained in the auditory cortex slices. For such auditory cortex slices, we recorded spatiotemporal population activity (local field potentials, LFPs) at multisites using a 64-channel multielectrode array (MEA) substrate in response to brief current pulses prior to and post-TS. Moreover, the TS to layer 4 of auditory cortex slices can possibly induce LTD at the electrode sites around the TS site. In fact, it has been reported that supragranular field potentials of rat slices, including the auditory cortex, are depressed after local TS at 100 Hz for 1 s applied to supragranular layers near the recording sites (Murakami et al., 2004; Watanabe et al., 2007). However, LTD criteria based on neural population activity (i.e., LFP peak intensity level) are not clearly distinguishable from the reduction of neural excitability in brain slices. Therefore, a convenient experimental method to examine the tetanic induction effect of LTD on neural excitability reduction is truly needed.

Here, after applying the TS to auditory cortex slices obtained from young adult mice (postnatal weeks 7–13), we first examined the occurrence of LTP and/or LTD. We found that only LTD was always observed in our stimulation condition. Next, to characterize the LTD properties of auditory cortex slices, we propose a recording protocol while simultaneously monitoring the activity levels at many recording sites [inter-electrode distance (IED), 0.15 mm], including the sites at more than 0.45 mm away from a tetanic stimulation site. In this study, our aim is to characterize LTD properties and distinguish LTD-induced reductions in neural excitability from other types, including neural activity depletion. To this end, we carefully monitored the LFP levels simultaneously at the many sites near (IED ≤ 0.45 mm) and away (IED > 0.45 mm) from a TS site. Accordingly, the reduction of neural excitability in a brain slice can be easily checked by examining if the level of test stimulus-evoked activity at the recording sites away from the stimulation remains unchanged or not during a series of experiments. Thus, our experimental protocol can distinguish the reduction of neural excitability induced by LTD from other types of excitability reductions over whole brain slices. In addition, to understand the sensitivity of synaptic plasticity to neurotransmitters, we applied an agonist or one of several antagonists for synaptic receptors, including the receptors of NMDA and GABA, to the extracellular perfusion solution. Furthermore, this study explores key candidates of synaptic receptors associated with synaptic plasticity on the basis of electric response kinetics, combining the results of electrophysiological experiments with a simple mathematical model. We hypothesize that transient LTD responses after TS can be described by a simple dynamical model with monostable or bistable equilibrium properties. In particular, we applied the modified version of a simple synaptic model proposed by Graupner and Brunel (2012) to the plastic responses in the dynamics of neural activity measured using brain slices. Thus, the model coefficients characterizing the response dynamics were estimated depending on the experimental conditions under the inhibition or prompting of synaptic transmission. Using the estimated model coefficients, we searched for key receptors involved in synaptic plasticity and quantitatively clarified their effects on response dynamics. Using these approaches, we propose a convincing method for LTP/LTD experiments and examine synaptic plasticity of cortico-cortical connections in the matured mouse auditory cortex beyond the auditory critical period, expecting a future application of brain stimulation as the medical treatment of hearing disorders.

## 2 Materials and methods

### 2.1 Brain slice preparation

All animal experiments described below were carried out in accordance with the Unified and Detailed Guidelines of the Science Council of Japan for laboratory animal experiments and with the approval of the Institutional Animal Care and Use Committee of Hokkaido University.

**Figure 1:**
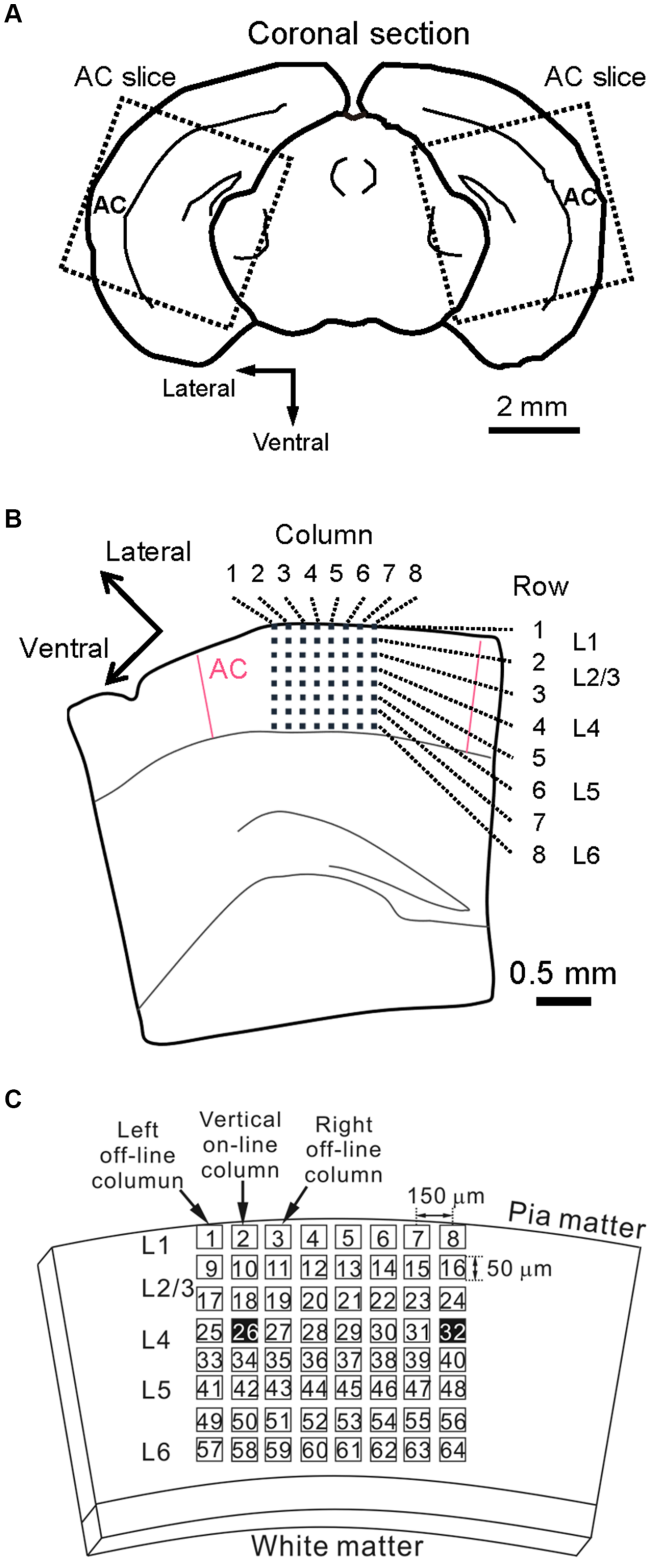
Schematic representation of a mouse brain slice including the auditory cortex and a multielectrode array (MEA) substrate. **(A)** Coronal slice sectioning in the mouse brain and brain slices (dotted squares). **(B)** A brain slice was placed on a two-dimensional (2D) MEA substrate. The six layers (L1–L6) of the cortical laminar structure are also illustrated. **(C)** Schematic drawing of a coronal slice on the MEA substrate. For example, the tetanic stimulus site (ch. 26) is indicated as one of the black squares (left black square). To monitor the excitability of each brain slice over 40 min, an additional electrode site was selected (ch. 32, right black square), and test stimuli were alternatively applied from the two sites during the experimental session.

To record LFPs from mouse brain slices *in vitro*, 400-μm-thick coronal slices including the auditory cortex (Figure 1A) were prepared from 7- to 13-week-old C57BL/6 J mice (Japan SLC Inc., Japan). The average postnatal week of the mice was 10.7 ± 0.4 (mean ± standard error of the mean (SEM); *n* = 23 animals). In this study, the distance of each coronal section from the bregma along the rostral/caudal axis was the same as described in Refs (Yamamura et al., 2017; Yamamura and Tateno, 2017) and defined in the following way. Briefly, a digitized atlas (Franklin and Paxinos, 2008) provided illustrations of coronal sections through the mouse brain. For coronal slices, we chose the coronal section that best matched the illustration at 2.92 mm caudal to bregma [Figure 65 in Franklin and Paxinos (2008)]. For slicing a brain block, including the auditory cortex, chilled artificial cerebrospinal fluid (ACSF) saturated with 95% O_2_ and 5% CO_2_ mixed gas was prepared. The ACSF contained (in mM) 119 NaCl, 2.5 KCl, 2.5 CaCl_2_, 1.3 MgSO_4_, 1.0 NaH_2_PO_4_, and 11.0 D-glucose (pH = 7.4). A mouse was deeply anesthetized with isoflurane (Viatris Inc., Pennsylvania, United States) and decapitated. Slices were then cut with a tissue slicer (Linear Slicer Pro7, D.S.K., Japan) in the chilled ACSF. The slices were recovered in a submerged-type holding chamber at 28°C in a water bath for at least 2 h before recording.

### 2.2 Electrophysiological recording using a multielectrode array substrate

In our experiments, all electrophysiological recording in brain slices was performed with perfusion of the ACSF solution and a mixed gas supply from the top of the recording chamber in an incubator (APC-30, Asteck Co., Japan) maintained at 28.0°C. While recording, slices were plated on multielectrode substrates (MED-P515A, Alpha MED Scientific, Japan) and covered with a nylon mesh and a stainless slice anchor. Each of the 64 recording sites (or channels; chs.) in the array covered an area of 50 × 50 μm^2^, and the IED between the centers of adjacent sites was 150 μm. The arrangement details between an electrode array and a brain slice, including the auditory cortex, were described in our previous report (Yamamura et al., 2017). Briefly, the top electrode row on each electrode array substrate was located at cortical layer 1 and paralleled with the brain surface. In the arrangement, the fourth row from the top was located in layer 4, or the border of layers 4 and 5 (Figures 1A,B).

### 2.3 Electrical stimulation and recording protocol

**Figure 2:**
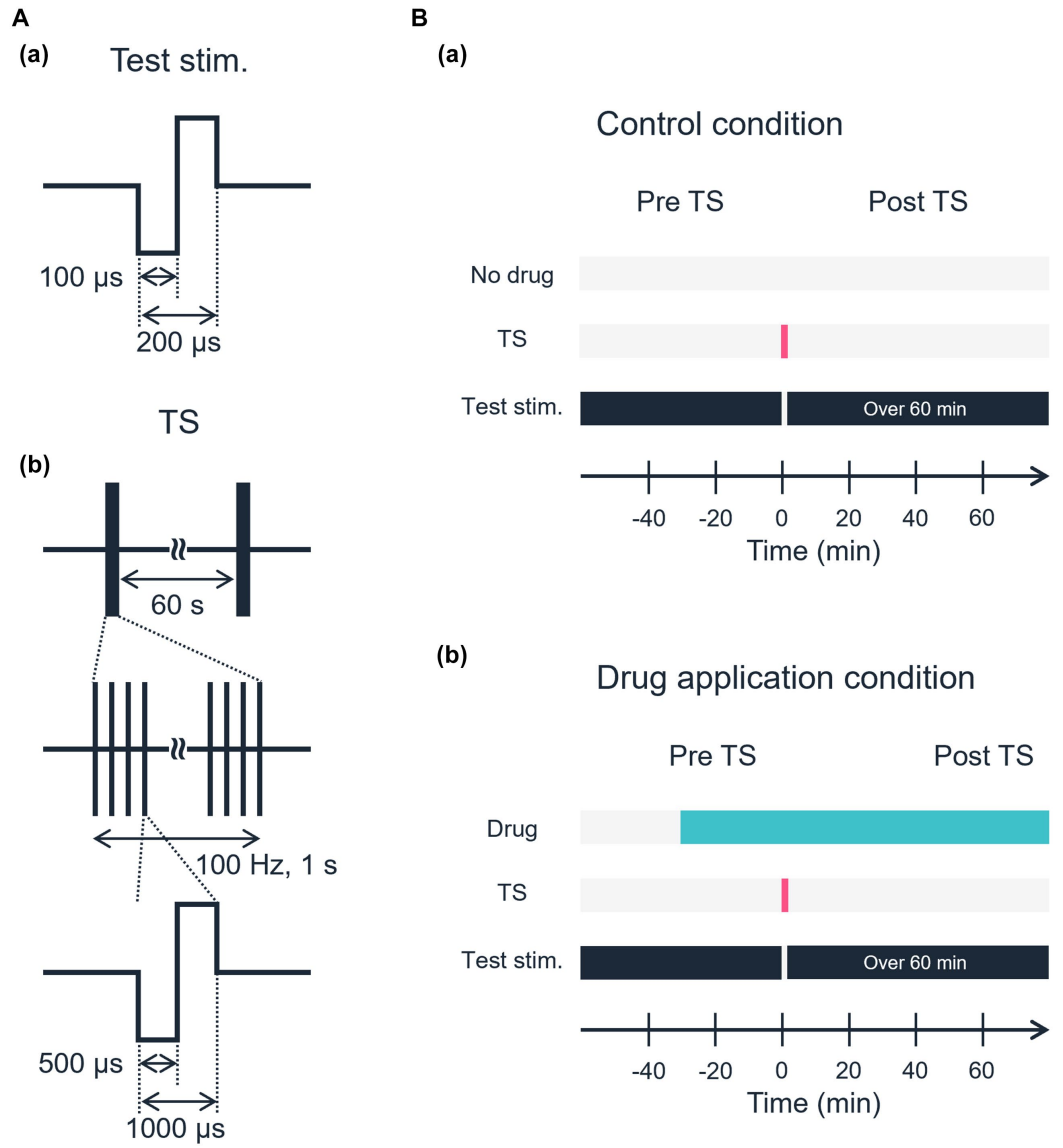
Schematic of the electrical stimulation protocol. **(A)** Waveforms of electrical stimulation. **(a)** Test stimulation. **(b)** Tetanic stimulation. **(B)** Experimental time sequences. **(a)** Control condition. No drug was applied in the control condition. **(b)** A specific drug was applied, and stable responses induced by test stimulation were confirmed before TS.

In each experiment, two stimulation electrode sites were first selected (i) one site for both TS and test stimulation and (ii) the other site for test stimulation only. Thus, both electrode sites were located in layer 4 or the border between layers 4 and 5 (Figures 1A,B), and the distance between the electrode centers was 750 μm away or more. The two sites were selected on the same row of the electrode array to monitor the excitability of the tested slices over a series of stimuli. Each experiment consisted of three stimulation sessions: (i) a pre-TS session, (ii) a TS session, and (iii) a post-TS session. In the pre-TS session, after confirming the stability of the responses, we applied test stimuli alternatively at the two sites every 10 s for 10–20 min to obtain baseline responses as control. In this study, the test stimulus was a single 200-μs-width bipolar pulse: 100 μs at negative current, followed by 100 μs at positive current. In the TS session, TS (100 Hz for 1 s, single 1,000-μs-width bipolar pulses; 500 μs at negative current followed by 500 μs at positive current) was applied to one of the stimulation sites, which is hereafter referred to as the TS site. The TS was applied two times at an interval of 60 s (Figure 2A; Watanabe et al., 2007). Although the TS was not directly applied at the other site, which is referred to as the non-TS site, the stimulation could slightly influence the activity of neurons around the non-TS site. In the post-TS session, we repeatedly applied test stimuli alternatively at the two sites every 10 s for over 40 min to examine the effects of TS. For a typical example, two stimulation sites in layer 4 were selected at sites 26 and 32, which were TS and non-TS sites, respectively (Figure 1C); the IEI of the two sites was 900 μm. The total recording duration in the typical experiment was more than 50 min, including the TS.

Throughout the experiments described here, we used a fixed small stimulation current intensity of 25 or 30 μA. The intensity was usually <30% of the saturation level in the input–output relationship between input current intensities and output-evoked LFPs (Namikawa et al., 2017; Yamamura et al., 2017). Thus, throughout all experiments, we carefully determined stimulation levels and monitored the activity responses driven from both the TS site (e.g., ch. 26 in Figure 1C) and non-TS site (ch. 32 in Figure 1C) before obtaining the main results of the post-tetanic session for individual slices. Typically, the intensity (*I*_tetanus_) of TS current was three times greater than that of test stimulation current, i.e., *I*_tetanus_ = 75 or 90 μA.

The locations of all recording positions on the multielectrode substrate were digitally imaged and recorded at the time of the experiment and histologically correlated with the laminar positions. After each experiment, current source density patterns (source and sink positions) were used to confirm the cortical layers by calculating the second spatial derivative of average LFP along vertical electrode sites (the column of the 2D array; data not shown in this study). In the 2D array, a column with recording sites vertical to the TS site (e.g., ch. 26 in Figure 1C) is referred to as a vertical “on-line” column indicated by an arrow. Also, two columns with recording sites aside of the tetanic stimulus site are referred to as vertical “off-line” columns (Figure 1C).

### 2.4 Criterium of neural activity reduction and data analysis

In this study, once the post-tetanic activity level stimulated from a non-TS site was reduced to 70% of the pre-TS activity level, we discarded the recording data from our result because the TS with the above-mentioned current intensity normally does not influence the activity level stimulated from the non-tetanic test site. Moreover, to analyze laminar response profiles, we performed a current source density analysis. Then, the cortical layers were determined on the basis of the distance from the brain surface and current source density profiles, following our previous method described Yamamura et al. (2017) and Yamamura and Tateno (2017). In the following, we particularly focus on neural activity on electrode sites on the two vertical columns (left- and right-off-site columns in Figure 1C) just adjacent to the TS on-site column, in which the TS was applied to one site in layer 4. Additionally, the selected non-TS site was also given special attention to monitor the reduction of slice excitability.

Here, to characterize LFP changes induced by the TS, we used a ratio index (RI) calculated from LFP recording data as usual. When LFPs were stable (i.e., amplitude variations less than 15%) during the pre-TS session over 10 min and the post-TS session over 40 min, for example, the magnitudes of negative peak LFPs 5 min prior and 40 min posterior to the TS are denoted by *V*_pre_(05) and *V*_post_(40), respectively. Then, the ratio was calculated by *V*_post_(40) over *V*_pre_(05), and the RI in percentage was defined as RI (40 min) = *V*_post_(40)/*V*_pre_(05). To illustrate LFP waveforms, successive five LFP trances in the same condition were averaged to reduce unexpected noise during recording.

### 2.5 Administration of agonist/antagonists for synaptic receptors

Furthermore, to understand the sensitivity of plasticity to neurotransmitters, we applied one of several drugs (agonist/antagonists) to the extracellular perfusion solution (Figure 2B). After stable baseline recording had been acquired for longer than 10 min by test stimulation before TS, we administered, at the end of each experiment, one of the following drugs: (i) α-amino-3-hydroxy-5-methyl-4-isoxazolepropionic acid (AMPA) and kainate receptor antagonist DNQX (Tocris Bioscience, Bristol, United Kingdom) at concentrations of 10 μM, (ii) NMDA receptor antagonist D-2-amino-5-phosphonopentanoic acid (D-AP5, Tocris Bioscience) at concentrations of 50 μM, (iii) metabotropic glutamate receptor 5 antagonist 6-methyl-2-(phenylethynyl)-pyridine hydrochloride (MPEP hydrochloride, Tocris Bioscience) at concentrations of 10 μM, (iv) GABA_A_ receptor antagonist bicuculline methiodide (Tocris Bioscience) at concentrations of 5 μM, (v) GABA_A_ receptor agonist muscimol (Toronto Research Chemicals, Toronto, CAN) at concentration of 5 μM, and (vi) GABA_B_ receptor antagonist CGP 54626 hydrochloride (Tocris Bioscience) at 5 μM. Our selection of the drugs was based on the previous experimental reports on synaptic plasticity in the rodent cortex (Kudoh and Shibuki, 1994; Murakami et al., 2004; Watanabe et al., 2007). Furthermore, because GABA_A_ receptor antagonists reduce the inhibition of neural excitation, the frequent emergence of spontaneous overactivity, including repetitive burst activity, can interrupt typical evoked responses to the test stimulation. This results in a reduction of the evoked responses to the test stimulation. Otherwise, under the application of the GABA_A_ receptor antagonist, the evoked LFP responses took longer to return to baseline voltage compared to the control condition. Therefore, we applied an agonist to investigate the effect of GABA_A_ receptors on LTD. In contrast, compared to the GABA_A_ receptor antagonist, the GABA_B_ receptor antagonist as well as the glutamate receptor antagonists did not profoundly change the neural responses, so we did not examine the effects of agonists for these receptors. Data are based on experiments in 47 auditory cortex slices from 23 C57BL/6 J mice. The number of slices used for drug application experiments was as follows: (i) *n* = 7 slices for DNQX, (ii) 6 for D-AP5, (iii) 6 for MPEP hydrochloride (MPEP), (iv) 6 for bicuculline methiodide (BIC), (v) 8 for muscimol, (vi) 7 for CGP 54626 hydrochloride (CGP), and (vii) 7 for control. In summary, see Table 1.

**Table 1:** Drug (agonist/antagonists) conditions applied on electrophysiological recordings.

i	Drug	Neurotransmitter	Receptor subtype	Effect	Number of slices
1	D-AP5	Glu*	NMDA	Antagonist	6
2	DNQX	Glu	AMPA	Antagonist	7
3	MPEP	Glu	mGlu5	Antagonist	6
4	Bicuculline	GABA**	GABA_A_	Antagonist	6
5	CGP	GABA	GABA_B_	Antagonist	7
6	Muscimol	GABA	GABA_A_	Agonist	8

*Glu represents glutamatergic condition. **GABA indicates GABAergic condition.

### 2.6 A phenomenological model describing synaptic plasticity

We modeled synaptic plasticity on the basis of the calcium-based formalism proposed by Graupner and Brunel (2012). In the original model by Graupner and Brunel (2012), the dynamics of a synaptic efficacy variable *ρ* is described as


(1) $$\begin{array}{l} \frac{d\rho }{dt}=[-\rho \left(1-\rho \right)\left(\rho_{*}-\rho \right)+\gamma_{p}\left(1-\rho \right)\Theta \left(c\left(t\right)-\theta_{p}\right)\\ \;-\gamma_{d}\rho \Theta \left(c\left(t\right)-\theta_{d}\right)+\text{Noise}\left(t\right)]/\tau , \end{array}$$

where *γ_p_* and *γ_d_* are the potentiation and depression rates, respectively. Θ is the Heaviside function, *θ_p_* and *θ_d_* are the potentiation and depression thresholds, respectively, *c*(*t*) is the intracellular calcium concentration, and *τ* is a time constant. Here, if *θ_p_* = 0, *θ_d_* = 0, and the noise term [i.e., Noise (*t*)] is neglected, the synaptic efficacy *ρ* = ρ_*_ (0 < *ρ* < 1) of Equation 1 determines the basin of attraction of the two stable states; each area is attracted to either of corresponding stable equilibrium points (*ρ* = 0 or *ρ* = 1) on potentiation or depression. Although the original model by Graupner and Brunel (2012) is a calcium-based model of a single synapse submitted to pre- and postsynaptic action potentials, we modified the model to apply it to evoked LFP responses of brain slices, which are the extracellular population responses including pre- and postsynaptic activities and action potentials. In our model, furthermore, the noise term in the original Graupner and Brunel model was removed because the time-averaged *ρ* during each interval of 10 min is considered. In addition, only LTD is hereafter considered, so that *γ_p_* is assumed to be zero; the state point *ρ*(*t*) is assumed to be stayed in the attraction bason concerning the depression. Furthermore, although *c*(*t*) is time-dependent in the original model, we assumed that $\Theta \left[c\left(t\right)-\theta_{d}\right]$ is time-independent beyond the early 10 min after administering TS. The essence of the model Equation 1 we used here is the bistable behaviors of the dynamical variable, which are consistent with some biochemical models based on physiological experiments (Zhabotinsky, 2000). Accordingly, in our model, the state variable *ρ* describes the dynamics as follows:


(2) $$\frac{d\rho }{dt}=\left[-\left(\rho -\alpha \right)\left(2-\rho \right)\left(\rho_{U}-\rho \right)-\gamma_{d}\left(\rho -\alpha \right)\right]/\tau ,$$

where the following condition should be satisfied:


(3) $$0<\alpha <\rho_{U}<2.$$

Although our model of Equation 2 is based on the Graupner and Brunel model (i.e., Equation 1), the synaptic efficacy variable *ρ* is shifted upward to +*α*. For easy understanding of LTD without normalization before TS, the lower stable point (*ρ* = *α*) concerning the depression is set to be 1.0 (i.e., *α* = 1.0) in our model, which does not critically affect the model dynamics after TS (Supplementary Figure 1A). Additionally, a potential function *V*(*ρ*) associated with Equation 2 is defined as


(4) $$V\left(\rho \right)=-\int_{0}^{\rho }f\left(u\right)du\;,$$

where $f\left(\rho \right)=\left[-\left(\rho -\alpha \right)\left(2-\rho \right)\left(\rho_{U}-\rho \right)-\gamma_{d}\left(\rho -\alpha \right)\right]/\tau$. For example, typical potential function *V* is illustrated in Supplementary Figure 1B. Furthermore, the following quadratic equation:


(5) $$\rho^{2}-\left(2+\rho_{U}\right)\rho +2\rho_{U}+\gamma_{d}=0,$$

gives a discriminant *D* described as


(6) $$D={\left(2-\rho_{U}\right)}^{2}-4\gamma_{d}\;\text{.}$$

The number of stable points is classified by the sign of the discriminant *D*, where (i) D>0 and (ii) D≤0 indicate that the dynamics has two stable points and a single stable point, respectively. If D>0, Equation 5 has two solutions ($\rho =\rho_{+}$ and $\rho =\rho_{-}$), which are expressed as follows:


(7) $$\rho_{\pm }=\frac{1}{2}\left\{\left(2+\rho_{U}\right)\pm \sqrt{{\left(2-\rho_{U}\right)}^{2}-4\gamma_{d}}\right\},$$

and $\rho =\rho_{-}$ ($\alpha <\rho_{-}\leq \rho_{+}$) is the unstable point separating the two basins of attraction to the two stable points ($\rho =\rho_{+}$ and ρ=α). In this case, we assume that the minimum potential at larger $\rho =\rho_{+}$ corresponds to settle to LTP, although LTP is not treated by selecting an appropriate initial point beyond the attracting bason associated with $\rho =\rho_{+}$. On the other hand, the convergence of the state point to smaller ρ=α is assumed with LTD. In contrast, if D≤0, the system has only one stable point ρ=α, which is associated with LTD. The typical trajectories of the dynamics in our simplified model are shown in Supplementary Figure 1C.

### 2.7 Fitting the model coefficients, trajectory classification, and parameter analysis

From the time courses of RI transients during depression recovery processes in obtained data sets [i.e., six points of RI(*t*) where *t* = 10, 20, …, 60 (min)], we determined five parameters: *ρ*_0_, *α*, *ρ_U_*, *γ_d_*, and *τ*. First, because *ρ*_0_ is the initial state point, we assumed that *ρ*_0_ = RI (10). Subsequently, the α, τ, $\gamma_{d}$ and $\rho_{U}$ were fitted to experimental data in a parameter space indicated in Table 2. The five parameters were optimized for seven groups at eight electrode sites, i.e., 56 (= 7 × 8) sets of the experimental data. In the original model by Graupner and Brunel (2012), in response to presynaptic action potential input into a postsynaptic neuron, synaptic activity induces fast changes within milliseconds in the efficacy variable. In the absence of the presynaptic activity, the postsynaptic activity slowly decays to one of the stable steady states on a time scale of minutes. Therefore, our model of Equation 2 can mainly mimic the slow dynamics of the original model of Equation 1, although the balance between the fast and slow dynamics was incorporated in the original differential equation.

**Table 2:** Parameters used in fitting to the synaptic model.

Parameter	Range	Step
α	0.1 to 0.6	0.05
τ	1 to 3600	300
$\gamma_{d}$	0.1 to 6.0	0.1
$\rho_{U}$	α to 2.0	0.1

Furthermore, eight channels were chosen from eight sites around the tetanic stimulation site (c.f., Figure 1C). Here, to estimate the coefficients of the model parameters, we define an error function (EF) described as


(8) $$EF=\sum_{i=1}^{6}|\rho \left(10i\right)-RI\left(10i\right)|\;\;\left(i=1,\cdots ,6\right)\text{.}$$

For each data set, a set of parameters minimizing EF was selected by searching for an optimal parameter set in a grid of the parameter space (Table 2; Charnes et al., 1955).

After obtaining the parameter sets of the model in Equation 2, we simulated trajectories of the model by numerically solving the differential equation (Equation 2) with the initial value [i.e., *ρ*_0_ = RI (10)]. All trajectory data were subsequently projected into the reduced dimensional space using the principal component analysis (PCA). In the data reduction analysis, we thus constructed the two-dimensional space to classify the drug-conditioned groups. To classify the groups, we calculated the centroid vector ***c*** for each cluster. Next, we evaluated the relevant distances *d_i_* from the control cluster as follows:


(9) $$d_{i}=\sqrt{{\left(c_{i1}-c_{01}\right)}^{2}+{\left(c_{i2}-c_{02}\right)}^{2}},$$

where *i* (= 1, 2, …, 6) represents the conditioned groups excluding the control group (Table 1); *c*_01_ and *c*_02_ denote the first and second components of the centroid vector [i.e., (*c*_01_, *c*_02_)] in the control group, respectively. Additionally, the two components ($c_{i1}-c_{01}$, $c_{i2}-c_{02}$) in Equation 9 represent a relative centroid vector of each drug group from that of the control group. As usual, for non-zero row vectors ***x*** and ***y***, the angle (*θ*) between ***x*** and ***y*** is defined as $\theta =\cos^{-1}\left[xy^{t}/\sqrt{\left(xx^{t}\right)\;.\;\left(yy^{t}\right)}\right]$, where $a^{t}$ denotes the transpose of a vector *a*. Moreover, because we observed two major clusters: (i) glutamatergic cluster and (ii) GABAergic cluster in the two-dimensional space, the classification boundary was estimated by using support vector machine (SVM; Li et al., 2023). To classify parameter sets, we collected all parameter data sets and constructed new vectors ($q_{n}$) defined as


(10) $$q_{n}=\left[\alpha_{n},\gamma_{dn},\rho_{Un},\rho_{0n}\right],$$

where *n* is the number of data (i.e., *n* = 1, …, 56). In the vector components, because τ had relatively a larger value than those of other parameters, we excluded τ from the classification analysis. However, this exclusion did not cause major differences, because the time scale related to τ was similar among the data sets. Then, to assess the spatial variability in the parameter space, the standard division between the parameter sets was calculated among the eight channels.

### 2.8 Data analysis

For the support vector machine, we used the Python open-source library (scikit-learn, Python 3.11.1). Statistical analyses between two groups were performed using paired and unpaired *t*-tests for normally distributed data; otherwise, the Mann–Whitney *U* test was used. Statistical analyses for multiple comparisons involving multiple groups were performed using the Steel-Dwass test following an ANOVA (Matlab, 2022a, MathWorks, United States; Python Ver. 3.8.5). A value of *p* < 0.05 was considered to be significant. Data are expressed as mean ± standard error of the mean (SEM). Error bars represent SEM.

## 3 Results

### 3.1 Long-term depression induced by local tetanic stimulation

**Figure 3:**
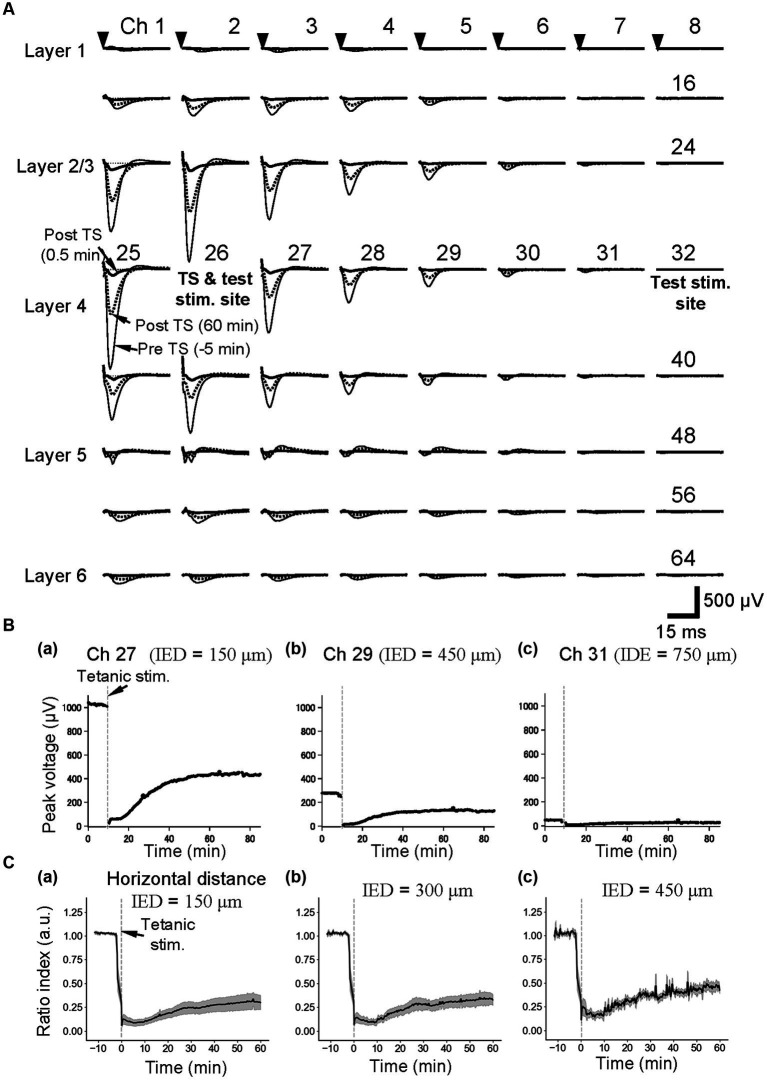
Current-evoked responses on an MEA substrate and the time courses of LTD before and after tetanic stimulation (TS). **(A)** In a slice of the mouse neocortex, evoked LFPs were extracellularly recorded prior to and posterior to TS on the MEA substrate. Thin, thick, and dotted lines, respectively, represent the LFP response waveforms at 5 min before tetanic stimulation (pre-TS, −5 min), immediately after the tetanic stimulation (post-TS, 0.5 min), and 60 min after the tetanic stimulation (post-TS, 60 min). Averaged LFP responses were evoked via current test stimulation at a single electrode site (ch. 26), which was in layer 4 and the same electrode to which the tetanic stimulation was applied. The stimulation current intensity was 25 μA. **(B)** For the horizontal three electrode sites to the stimulation site (ch. 26) under the same brain slice in A, the time courses of LTD for the tetanic stimulation applied at time 0 min are shown. The inter-electrode distances (IEDs) between the electrode site (ch. 26) of the test stimulation and each of the recording sites (chs. 27, 29, and 31) were 150, 450, and 750 μm, respectively. **(C)** As the control condition (normal extracellular medium), average time courses of LTD from group data (*n* = 5) were illustrated. Response peak amplitudes posterior to the TS were normalized by those prior to the TS. The IEDs between the electrode site (e.g., ch. 26) of the test stimulation and each of the recording sites are also indicated.

To examine a neural activity level prior to and post-TS, single short test pulses were alternatively delivered at low current intensities (25 or 30 μA) as stimuli from two single electrodes (e.g., ch. 26 or 32 in Figures 1C, 3A) to layer 4 of auditory cortex slices. In response to the stimulation at the single sites, large negative LFP responses with short latency were evoked at the surrounding sites of the stimulation sites (e.g., stimulation site at ch. 26 in Figure 3A). The maximum amplitudes of the LFP responses driven from the single sites in layer 4, which is one of the main recipient layers from the neurons in the ventral division of the medial geniculate body *in vivo*, were always observed at recording sites in layer 2/3 or layer 4 (Figure 3A). In contrast, positive-going responses in layer 5 can be observed in some recording channels, following negative-going responses with short latencies (e.g., chs. 42–45 in Figure 3A). In layer 4, particularly, typical evoked LFPs of auditory cortex slices during a 100-ms time window after the stimulation onset were composed of three components: (i) an early negative-going potential (component A) from the stimulation onset to approximately 3 ms; (ii) a late positive-going potential (component B) following component A; and (iii) a final smaller negative-going potential (component C), as previously reported (Yamamura et al., 2017). The peak amplitudes in layer 4 in the control groups were 0.81 ± 0.04 mV (*n* = 6), and the latencies of the peaks from the stimulation onset were 5.0 ± 0.18 ms (*n* = 6). Although the brain slices in this study did not include many axon terminals from the MGB, the results were consistent with cortical laminar responses to MGB stimulation in mouse auditory cortex slices (Cruikshank et al., 2002).

In addition, evoked LFP responses decreased monotonically with a horizontal distance from the stimulation site (e.g., chs. 26–31 of the fourth row from the top in the 2D array of Figure 3A). When the horizontal distance from the stimulation site in layer 4 was over 750 μm (IED = 5 × 150 μm), LFP responses at recording sites of the same row were hard to distinguish from the baseline noise (ch. 31 in Figures 3A,Bc). In Figures 3A,Bc, the LFP amplitudes of the responses were over 1.0 mV when the distance was within 150 μm, whereas only smaller LFP responses were recorded at a site more than 450 μm away (e.g., ch. 29), and no clear responses were observed at the site over 750-μm distance (ch. 31 in Figure 3A).

Furthermore, the time courses of LTD in layer 4 are shown at the three electrode sites in Figure 3B. The effects of local TS could be profoundly affected by the horizontal distance between the stimulating and recording electrodes in layer 4. When response electrodes were close to the TS site, the peak amplitude of post-TS responses was reduced to RI (5 min) = 0.09 ± 0.04 (*n* = 5), 5 min after TS and RI (60 min) = 0.30 ± 0.08 (Figure 3Ca). The post-TS spatial responses of LTD induced by local TS were superimposed to pre-tetanic responses in Figure 3A (dotted and thick lines). The LTD-induced spatial responses were stimulation site-dependent, although the properties of spatial responses 60 min after tetanic stimulation were almost in proportion to those of pre-TS spatial responses.

On the other hand, after local TS (e.g., at ch. 26) was applied at a distance over 750 μm from the recording sites (e.g., chs. 24 and 40), LFP responses elicited by local layer-4 test stimulation (ch. 32) were almost unchanged (Supplementary Figure 2), indicating that LTD induced by local TS did not reflect any specific changes to the brain slice area at the recording sites caused by local TS in this condition with the normal extracellular medium. However, it is noteworthy that relative representation of the amplitude changes indicates that in recording sites even at 750-μm distance away from the TS site, the detectable relatively small changes in response amplitudes can be seen in several channels (Figure 3Cc).

In the following plots for the series of experiments concerning LTD induction and drug application, responses at the adjacent electrode of the tetanic and test stimulation sites (a horizontal distance of 150 μm between the two electrode centers, i.e., IED = 150 μm) were typically illustrated to show representative results; the amplitude of LTD induced at this distance was stable and similar to those of LTD induced at a distance ranging from 150 to 600 μm.

**Figure 4:**
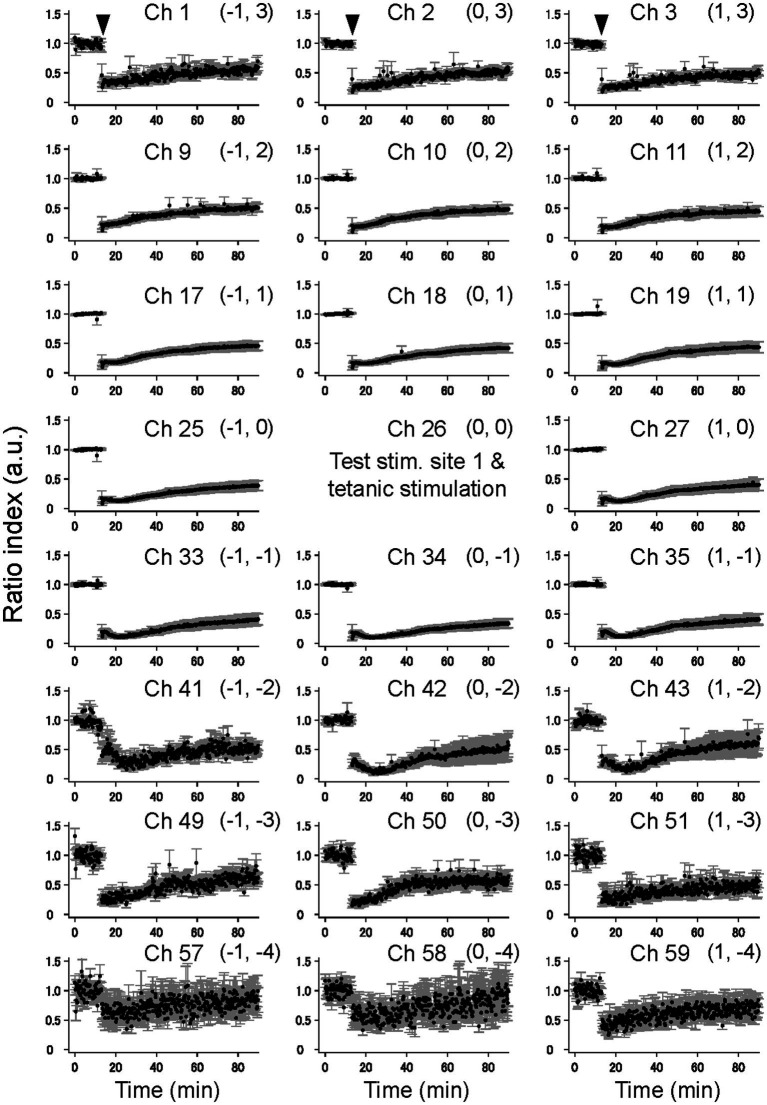
The time courses of LTD in the cortical laminar layers prior and posterior to TS. The average time courses of LTD for 23 electrode sites on vertical on-line and off-line columns (c.f., Figure 1C) are illustrated on average for five brain slices (*n* = 5). The tetanic and test stimulation sites are represented as ch. 26, which was the spatial reference point [i.e., the origin (0, 0)] in a two-dimensional array. To express each element in the 2D matrix in the array, a bracket representation (*a*, *b*) denotes an element in the 2D matrix, where *a* and *b*, respectively, represent the row and column number relative to the origin (0, 0). The time points of TS around 10 min are indicated by inverted triangles in the first row. The error bars represent the standard mean of errors (SEMs) of normalized LFP amplitudes among the samples at the same time points.

Moreover, elicited by test stimulation pulses at the tetanic stimulation site (e.g., ch. 26 in Figure 4), the vertical (laminar) LFP changes of the pre- and post-TS amplitudes are shown only for three columns of a 64-channel electrode array in five slices. On the top right in each panel of Figure 4, a pair of numbers in a bracket represents a relative position to the stimulation site. For example, (1, 1) indicates ch. 19, which is the upper right to the stimulation site (0, 0). In all electrode sites in Figure 4, normalized LFP responses excluding several channels were reduced after TS, although LFP responses in the bottom electrodes were not significantly changed (chs. 57, 58, and 59 of Figure 4).

In addition, elicited by test stimuli at the other stimulation site (ch. 32 in Figure 3A), 900 μm away from both the tetanic and test stimulation sites (ch. 26 in Figure 3A), all LFP responses in the electrode sites of the three columns did not significantly change (Supplementary Figure 2) and thus showed no LTD in cortical laminar layers. The results indicate that the LFP response levels in these laminar layers were maintained during the experimental period, which lasted over 80 min from the beginning of the recording.

### 3.2 Effects of glutamate receptor antagonists on response waveforms and LTD

Next, to understand how pharmacological antagonism of glutamate receptors characteristically modifies the transient properties of LTD induced by TS, we examined the effects of glutamate receptor antagonists using the same experimental setup (Supplementary Figure 3). During application of an NMDA receptor antagonist (50-μM D-AP5), the time course of amplitude changes of early negative-going component A indicated that D-AP5 did not substantially affect the component in the evoked LFPs obtained in layer 4 (e.g., 20–30 min after the onset of D-AP5 in Supplementary Figure 3Aa) from the recording sites adjacent to the test stimulation site in layer 4 vs. pre-application period (−10 to 0 min in Supplementary Figure 3Ab): 531.5 ± 216.3 μV vs. 867.9 ± 155.8 μV, respectively (*p* = 0.128, *n* = 6). Similarly, at recording sites in layer 4 (e.g., ch. 31 in Figure 3A; distance between the three electrodes, IED = 450 μm) far from the stimulation electrode (e.g., ch. 26 in Figure 3A), the peak amplitude (component A) of LFP responses (85.8 ± 36.1 μV; *n* = 6) was not significantly changed after application of D-AP5 (61.5 ± 30.5 μV; *p* = 0.689). Furthermore, in layers 2/3 and 5/6, the D-AP5 application affected the average negative-going component and reduced the average amplitude slightly, although no significant changes were found. Furthermore, the application of non-NMDA (AMPA/kainate) receptor antagonist (10-μM DNQX) reduced the early component A (189.6 ± 80.4 μV vs. 96.6 ± 46.4 μV; *p* = 0.250; *n* = 7), suggesting that LFP responses were mainly mediated by non-NMDA receptors (Supplementary Figure 3B). Also, the positive-going component B was slightly reduced, resulting in no crossing over the baseline at 0 V after the DNQX application. Additionally, administering 10-μM MPEP, an antagonist of metabotropic glutamate receptors, was not effective to change the response waveforms driven by the test stimulation to layer 4 (Supplementary Figure 3Ca): the early component A (394.4 ± 108.3 μV vs. 275.4 ± 154.0 μV; *p* = 0.403; *n* = 5).

**Figure 5:**
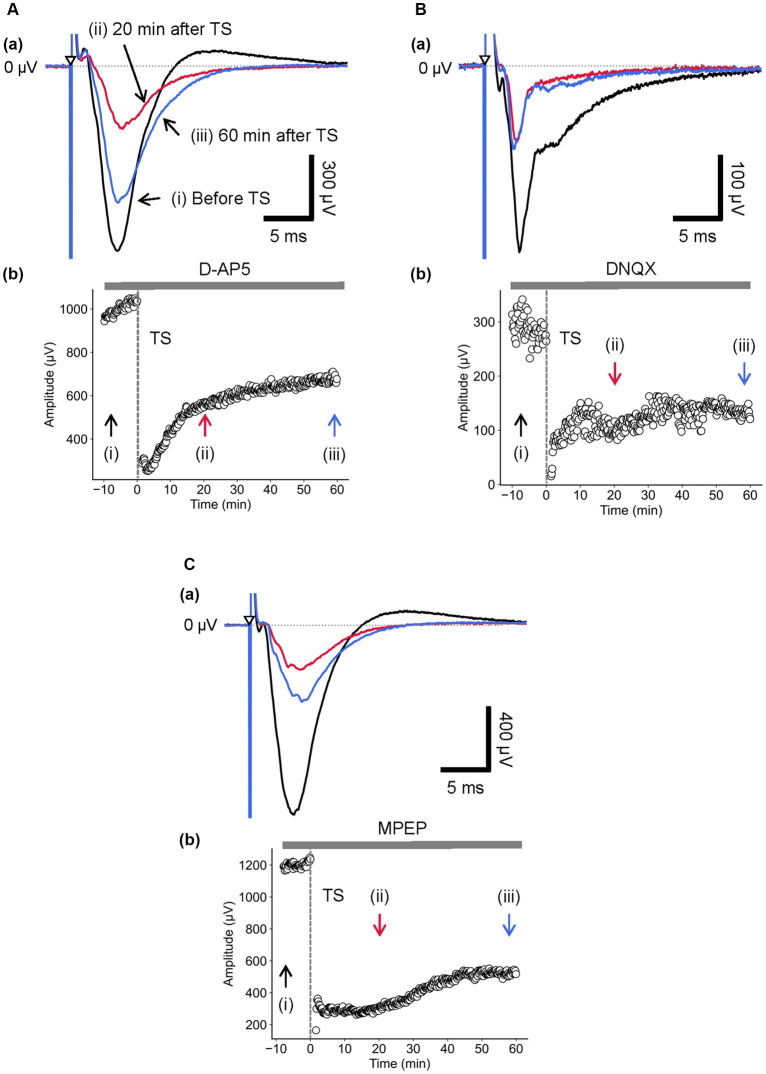
Current-driven responses in layer 4 of a brain slice under the glutamatergic receptor antagonist/agonist condition. **(A)** Under 10-μM D-AP5 application, LFP responses were recorded in layer 4 before and after TS. Three LFP responses are illustrated (i) before (black trace), (ii) 20 min (red trace), and (iii) 60 min after TS (blue trace) applied to layer 4 in **(a)**. The test stimuli were repetitively applied to layer 4. Time course of negative-going peak magnitude is illustrated during the whole recording period (70 min) in **(b)**. **(B)** Similarly, under 50-μM DNQX application, LFP responses were recorded in layer 4 before and after TS. Three LFP responses are illustrated (i) before (black trace), (ii) 20 min (red trace), and (iii) 60 min after TS (blue trace) applied to layer 4 in **(a)**. Time course of negative-going peak amplitudes is illustrated during the whole recording period (70 min) in **(b)**. **(C)** Under 50-μM MPEP application, LFP responses were recorded in layer 4 before and after TS. Three LFP responses are illustrated (i) before (black trace), (ii) 20 min (red trace), and (iii) 60 min after TS (blue trace) applied to layer 4 in **(a)**. The test stimuli were repetitively applied to layer 4. Time course of negative-going peak amplitudes is illustrated during the whole recording period (70 min) in **(b)**. TS was applied at time 0 min in the plot (indicated by a dashed line and an arrow). The timings of a test stimulus are indicated by the inverted triangles in **(a)**.

During the D-AP5 application, LTD in layer 4 was induced by TS applied to the same layer. As shown in Figure 5A, the amplitude of the early negative-going component was significantly reduced [RI (5 min) = 0.21 ± 0.03, *p* = 0.005; *n* = 6], and the late positive-going component was also decreased (Figure 5Aa). Figure 5Ab shows the time course of response amplitudes in LTD. The depression was maximized immediately after the TS, gradually recovered during 10–20 min after the TS, and the response level was maintained thereafter over 40 min. The amplitude of LTD measured 40 min after LFS was RI (40 min) = 0.41 ± 0.18 of the baselines before TS and significantly decreased compared to the baselines (Figure 5Ab; *p* = 0.005, *n* = 6). Additionally, Figure 5B shows the time course of response amplitudes in LTD for the application of non-NMDA (AMPA/kainate) receptor antagonist (10-μM DNQX). During the DNQX application, LTD in layer 4 was induced by TS applied to the same layer. As shown in Figure 5B, the amplitude of the early negative-going component was significantly reduced [RI (5 min) = 0.38 ± 0.15, *p* = 0.029; *n* = 7], and the recovering phase to the baseline was also decreased (Figure 5Ba). The depression was induced immediately after the TS, slightly increased during 20–30 min after the tetanic stimulation, and the response level was maintained thereafter over 60 min. The amplitude of LTD measured 40 min after LFS was RI (40 min) = 0.29 ± 0.14 of the baselines before the TS and significantly decreased compared to the baseline (*p* = 0.002, *n* = 7; Figure 5Bb).

**Figure 6:**
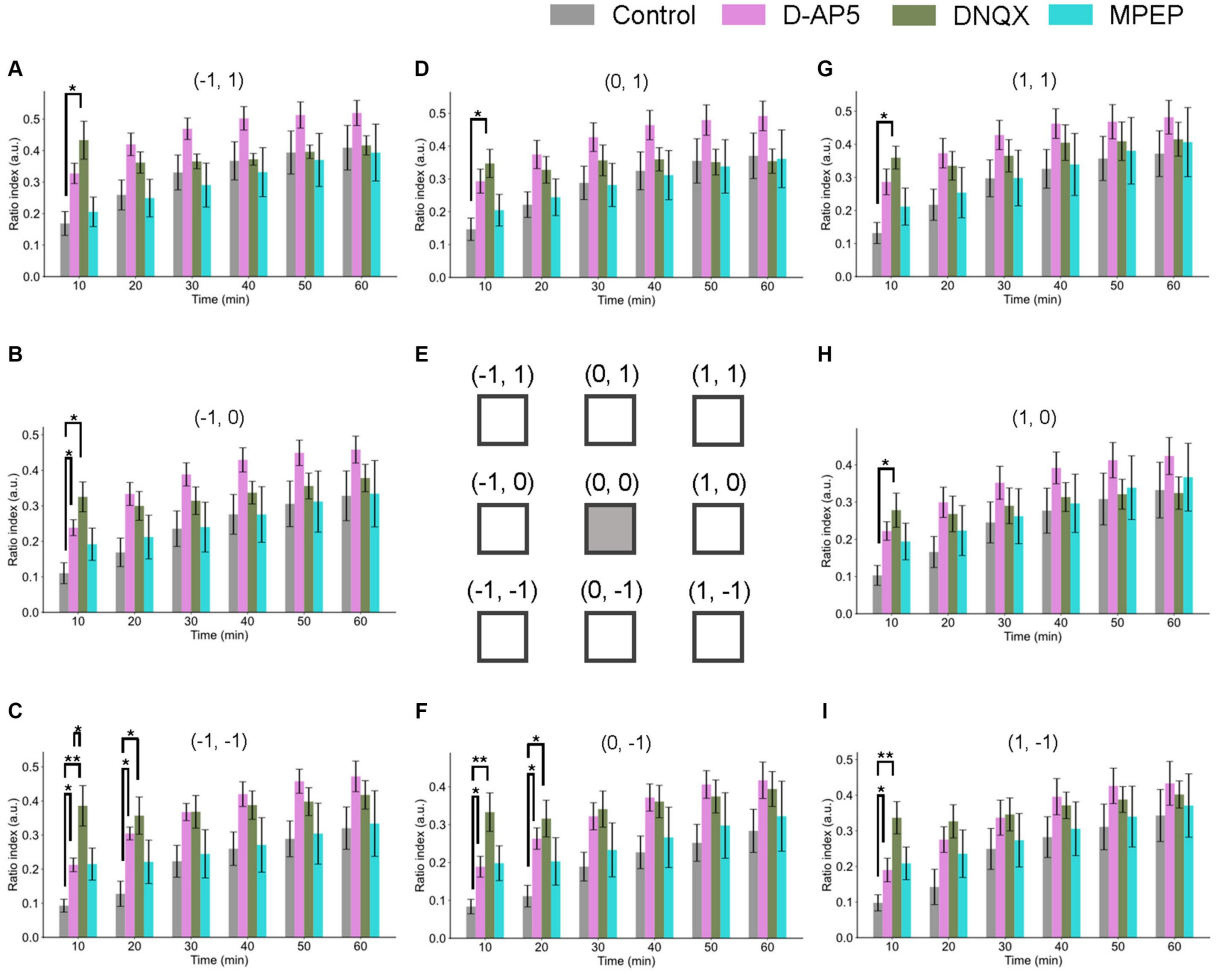
Spatiotemporal properties of LTD around the tetanic and test stimulation electrodes under the conditions of NMDA receptor, non-NMDA receptor, and metabotropic glutamate receptor 3 antagonists. Ratio indices (RIs) of evoked responses at eight recording sites around the TS site during 60 min posterior to TS under the control and the antagonists (10-μM D-AP5, 50-μM DNQX, and 50-μM MPEP) conditions. **(E)** schematic representation of the tetanic and test stimulation sites at position (0, 0) and the eight recording sites (−1, 1), (−1, 0), (−1, −1), (0, 1), (0, −1), (1, 1), (1, 0), and (1, −1) in a two-dimensional array representation for **(A–D,F–I)**, respectively. Each of the inter-electrode distances (IEDs) was 150 μm. In the abscissa of each bar graph, time in minutes represents the time points after TS with a 10-min step. We used an ANOVA for multiple comparisons followed by a *post-hoc* Steel-Dwass test: control (*n* = 7), D-AP5 (*n* = 6), DNQX (*n* = 7), and MPEP (*n* = 6). * and ** represent *p* < 0.05 and *p* < 0.01, respectively.

During the MPEP application, LTD in layer 4 was induced by TS applied to the same layer. The response amplitude of the early negative-going component A was significantly reduced (Figure 5Ca; RI (5 min) = 0.19 ± 0.05, *p* = 0.005; *n* = 6). Although the LFP amplitudes were reduced under the MPEP condition, the following positive-going LFP was monotonically approaching the baseline in the recovering phase without overshooting across the zero level (Figure 5Ca). Under the MPEP application, the depression of LFPs was induced immediately after the TS, and the LFPs were gradually increasing over 60 min. The amplitude of LTD measured 40 min after LFS was RI (40 min) = 0.32 ± 0.08 over the baseline before the TS and significantly decreased compared to the baseline (*p* = 0.005, n = 6; Figure 5Cb). In short summary, during the MPEP application, the spatial properties of induced LTD around the TS site are illustrated in Figure 6. The time course of LTD examined over 60 min under the MPEP application (*n* = 6) was compared to that of the control condition (*n* = 7). In all electrode sites under all conditions, LTD recorded at all eight sites was induced immediately after TS and slowly recovered over 60 min. The result indicates that the layer-dependency of LTD induced by TS was not found in the MPEP application, and metabotropic glutamate receptors contributed little to the LTD phenomenon described above.

The spatial properties of induced LTD around the TS site [represented by the 2D coordinate relative to the test and stimulation sites (0, 0)] are illustrated in Figure 6. The time courses of LTD examined over 60 min under D-AP5 (*n* = 6), DNQX (*n* = 7), or MPEP (*n* = 6) applications were compared to the control condition (*n* = 7). In all electrode sites under all conditions, the response suppression induced immediately after TS was recorded at all eight sites and maintained over 60 min (i.e., LTD). The amount of LTD and the time courses were different as a matter that depends on the antagonist conditions and the recording electrode site positions. Under the DNQX application for 10-min posterior to TS, the ratio indices [RI (10 min)] at all eight sites were significantly different from the control condition. Also, under the D-AP5 application for 10-min posterior to TS, RI (10 min) at site positions (−1, 0), (1, 0), (−1, −1), and (0, −1) were significantly different. However, in the next 10 min, although the tendency continued only in layer 5 and under D-AP5 and DNQX conditions, the differences were not significant under both antagonist conditions and in layers 2/3 and 4. The result indicates that LTD in infragranular layers was more prominently affected by the D-AP5 and DNQX applications via non-AMPA and non-NMDA receptors than other conditions. In short summary, LTD in all layers was induced under the conditions utilizing the glutamate receptor antagonists D-AP5 and DNQX. Furthermore, the LTD difference between the control condition and the two experimental conditions utilizing the glutamate receptor antagonists was limited to a short time period (duration of 10–20 min) after TS application.

### 3.3 Effects of GABAergic receptor agonist/antagonist on long-term depression

Inhibitory GABAergic synapses can play an important role to modulate neural activity as well as excitatory glutamatergic synapses. Here, we investigated the effects of antagonists of both GABA_A_ and GABA_B_ receptors and an agonist of GABA_A_ receptors on LTD induced by local TS to layer 4. With respect to the antagonist and agonist of GABA_A_ receptors, we used 5-μM bicuculline and 5-μM muscimol, respectively. In addition, as an GABA_B_ receptor antagonist, 5-μM CGP was used.

During the application of bicuculline and repetitive test stimulation to layer 4, the LFP amplitude of the early negative-going component A in layer 4 was not significantly changed compared to the absence of bicuculline (Supplementary Figure 4Aa; 559.2 ± 101.6 μV vs. 308.2 ± 91.8 μV, respectively; *p* = 0.128, *n* = 6). Therefore, the time course of amplitude changes of component A indicated that bicuculline did not affect the component in the evoked LFPs recorded in layer 4 (Supplementary Figure 4Ab).

**Figure 7:**
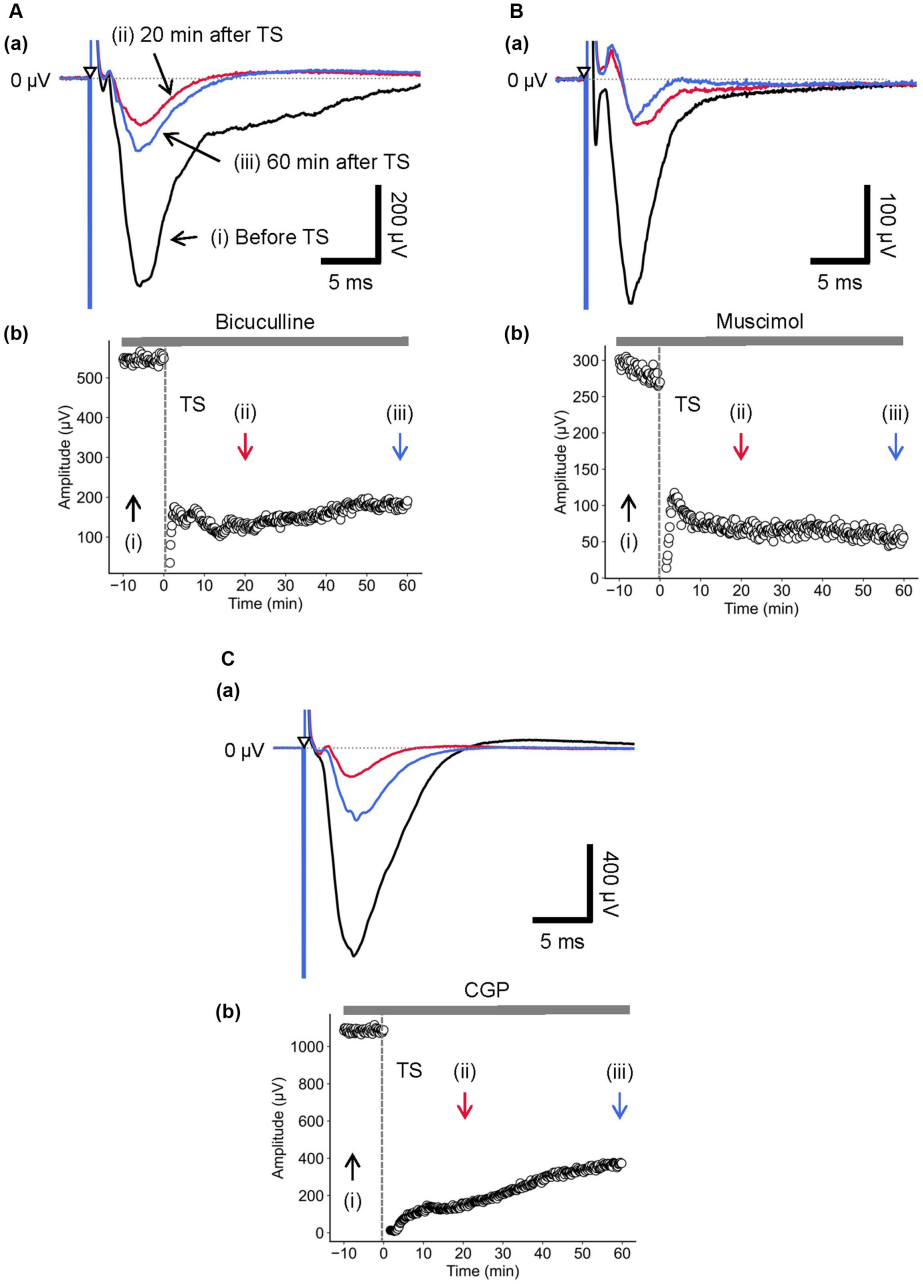
Current-driven responses in layer 4 of a brain slice under the conditions of GABA_A_ receptor antagonist/agonist and the GABA_B_ receptor antagonist. **(A)** Under 5-μM bicuculline application, LFP responses were recorded in layer 4 prior and posterior to TS. Three LFP responses are illustrated (i) before (black trace), (ii) 20 min (red trace), and (iii) 60 min after TS (blue trace) applied to layer 4 in **(a)**. The test stimuli were repetitively applied to layer 4. Time course of negative-going peak amplitudes is illustrated during the whole recording period (70 min) in **(b)**. **(B)** During 5-μM muscimol application, LFP responses were recorded in layer 4 prior and posterior to TS. Three LFP responses are illustrated (i) before (black trace), (ii) 20 min (red trace), and (iii) 60 min after TS (blue trace) applied to layer 4 in **(a)**. The test stimuli were repetitively applied to layer 4. Time course of negative-going peak amplitudes is illustrated during the whole recording period (70 min) in **(b)**. **(C)** Under 5-μM CGP application, LFP responses were recorded in layer 4 before and after TS. Three LFP responses are illustrated (i) before (black trace), (ii) 20 min (red trace), and (iii) 60 min after TS (blue trace) applied to layer 4 in **(a)**. The test stimuli were repetitively applied to layer 4. Time course of negative-going peak amplitudes is illustrated during the whole recording period (70 min) in **(b)**. TS was applied at time 0 min in the plot (indicated by a dashed line and an arrow). The timings of a test stimulus are indicated by the inverted triangles in **(a)**.

Next, during the bicuculline application, LTD in layer 4 was induced by TS applied to the same layer. As shown in Figure 7Aa, in the presence of bicuculline, the amplitude of component A was significantly reduced [RI (5 min) = 0.16 ± 0.04, *p* = 0.005; *n* = 6]. Furthermore, Figure 7Ab shows the time course of response amplitudes in LTD. The depression was induced immediately after the TS, and the reduced response levels were maintained thereafter for over 50 min. The amplitude of LTD measured 40 min after the local TS was RI (40 min) = 0.22 ± 0.03 over the baseline before the TS and significantly decreased compared to the baseline (Figure 7Ab; *p* = 0.005, *n* = 6).

Furthermore, during the application of muscimol and repetitive test stimulation to layer 4, the LFP amplitude of component A in layer 4 was first increased and then reduced gradually, resulting in significantly decreased amplitudes finally, compared to the absence of bicuculline (Supplementary Figure 4B; 555.6 ± 77.5 μV vs. 411.6 ± 51.8 μV, respectively; *p* = 0.041; *n* = 8). Therefore, the time course of amplitude changes of component A indicates that muscimol increased the LFP component for a short period and decreased thereafter in layer 4 over 30 min (Supplementary Figure 4Bb).

Additionally, during the muscimol application, LTD in layer 4 was also induced by TS applied to the same layer. In the presence of muscimol, the amplitude of component A was significantly reduced (Figure 7Ba; RI (5 min) = 0.37 ± 0.05, *p* = 0.001; *n* = 8). Similarly, Figure 7Bb shows the time course of response amplitudes in LTD. The depression was induced immediately after the TS, and the reduced response levels were maintained or continued to decrease thereafter over 50 min. These GABA_A_ receptor-related pharmacological experiments indicate that LTD induced by local TS in layer 4 is similar with regard to the sensitivity of both antagonist and agonist, implying that GABA_A_ receptors did not directly affect the LTD induction in the auditory cortex.

Similarly, during the application of CGP and repetitive test stimulation to layer 4, the LFP amplitude of component A in layer 4 was not significantly changed compared to the absence of CGP (Figure 7Ca; 625.6 ± 166.2 μV vs. 751.7 ± 230.8 μV, respectively; *p* = 0.665, *n* = 4). Therefore, the time course of amplitude changes of component A indicated that CGP did not affect the component in the evoked LFPs recorded in layer 4 (Figure 7Cb). During the CPG application, LTD in layer 4 was induced by TS applied to the same layer. As shown in Figure 7Ca, in the presence of CGP, the amplitude of component A was significantly reduced [RI (5 min) = 0.08 ± 0.05, *p* = 0.002; *n* = 7]. Furthermore, Figure 7Cb shows the time course of response amplitudes in LTD. The depression was induced immediately after the TS, and the reduced response levels were maintained thereafter for over 50 min. The amplitude of LTD measured 40 min after LFS was RI (40 min) = 0.17 ± 0.08 over the baseline before the TS and significantly decreased compared to the baseline (Figure 7Cb; *p* = 0.002, *n* = 7).

**Figure 8:**
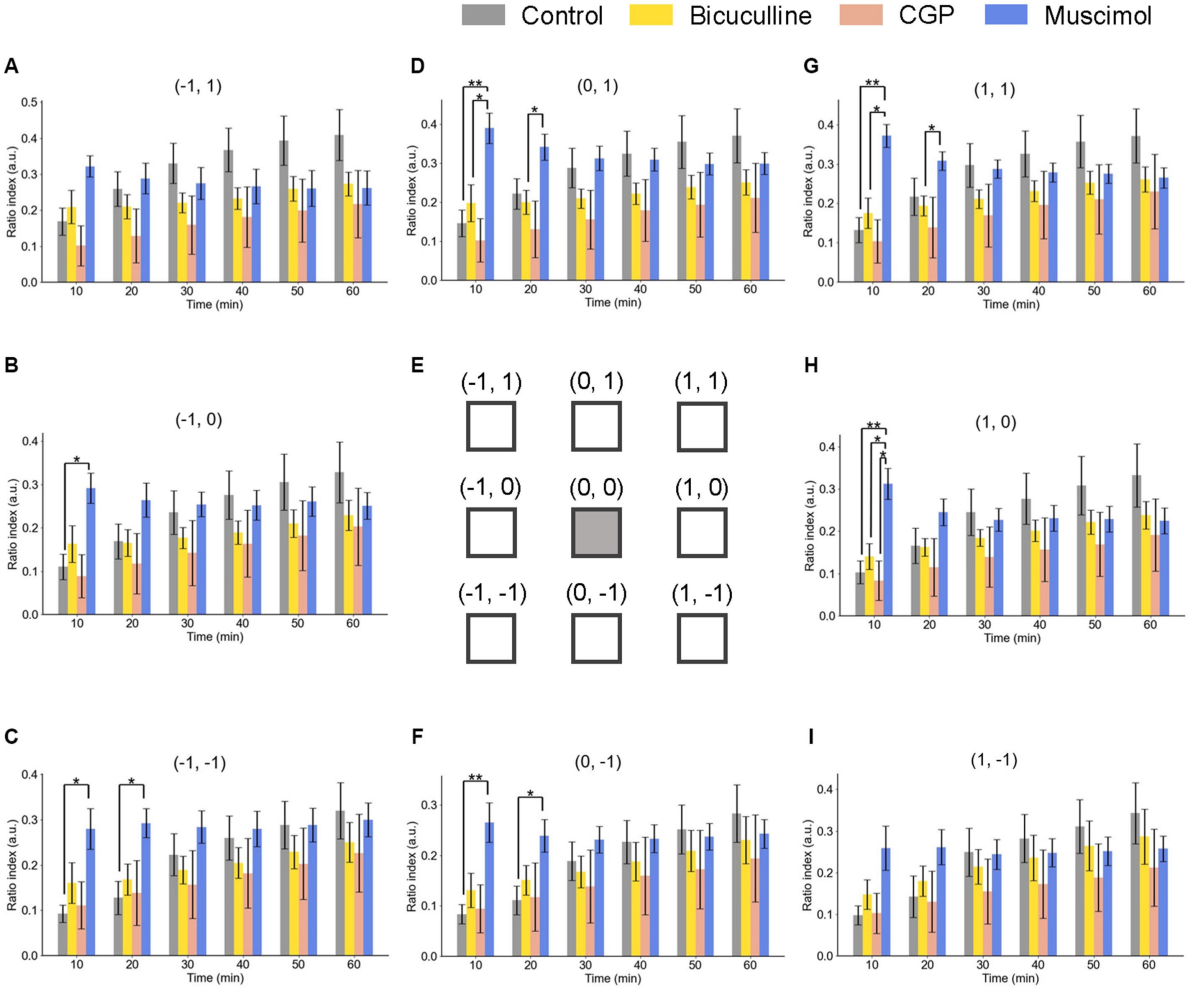
Spatiotemporal properties of LTD around the tetanic and test electrode sites under GABAergic receptor agonist/antagonist conditions. Ratio indices (RIs) of evoked responses at eight recording sites around the TS site during 60 min posterior to TS under the control and GABAergic antagonist (5-μM bicuculline and 5-μM CGP) and agonist (5-μM muscimol) conditions. The representation of Parts **(A–I)** is the same as that in Figure 6. We used an ANOVA for multiple comparisons followed by a *post-hoc* Steel-Dwass test: control (*n* = 7), bicuculline (*n* = 6), CGP (*n* = 7), and muscimol (*n* = 8). * and ** represent *p* < 0.05 and *p* < 0.01, respectively.

As a brief summary, under GABA receptor-related pharmacological conditions, the spatial properties of induced LTD around the TS site are illustrated in Figure 8 as a similar representation of Figure 6. The time courses of LTD examined over 60 min under bicuculline (*n* = 6), muscimol (*n* = 8), and CGP (*n* = 7) applications were compared to the control condition (*n* = 7). In all electrode sites under all conditions, LTD recorded at all eight sites was induced immediately after TS and maintained over 60 min. The amount of LTD and the time courses were different, as a matter that depends on the pharmacological conditions and the recording electrode site positions.

Under the muscimol application for 10-min posterior to TS, the ratio indices [RI (10 min)] at six electrode sites, excluding the top-left and bottom-right site positions (−1, 1) and (1, −1), respectively, were significantly different from the control condition. However, in the next several 10s of min, the significant differences were not maintained under the muscimol condition. The result indicates that LTD in all layers was not profoundly affected by GABA receptor-mediated pharmacological manipulation.

### 3.4 Analysis of the synaptic-efficient model under different drug conditions

Results from Figures 6, 8 show that the RI transients 10 min after TS and later differed among the drug conditions, suggesting the properties under the conditions can be characterized by the trajectories of the RI dynamics. Therefore, we applied the simple dynamical model to the RI data sets.

**Figure 9:**
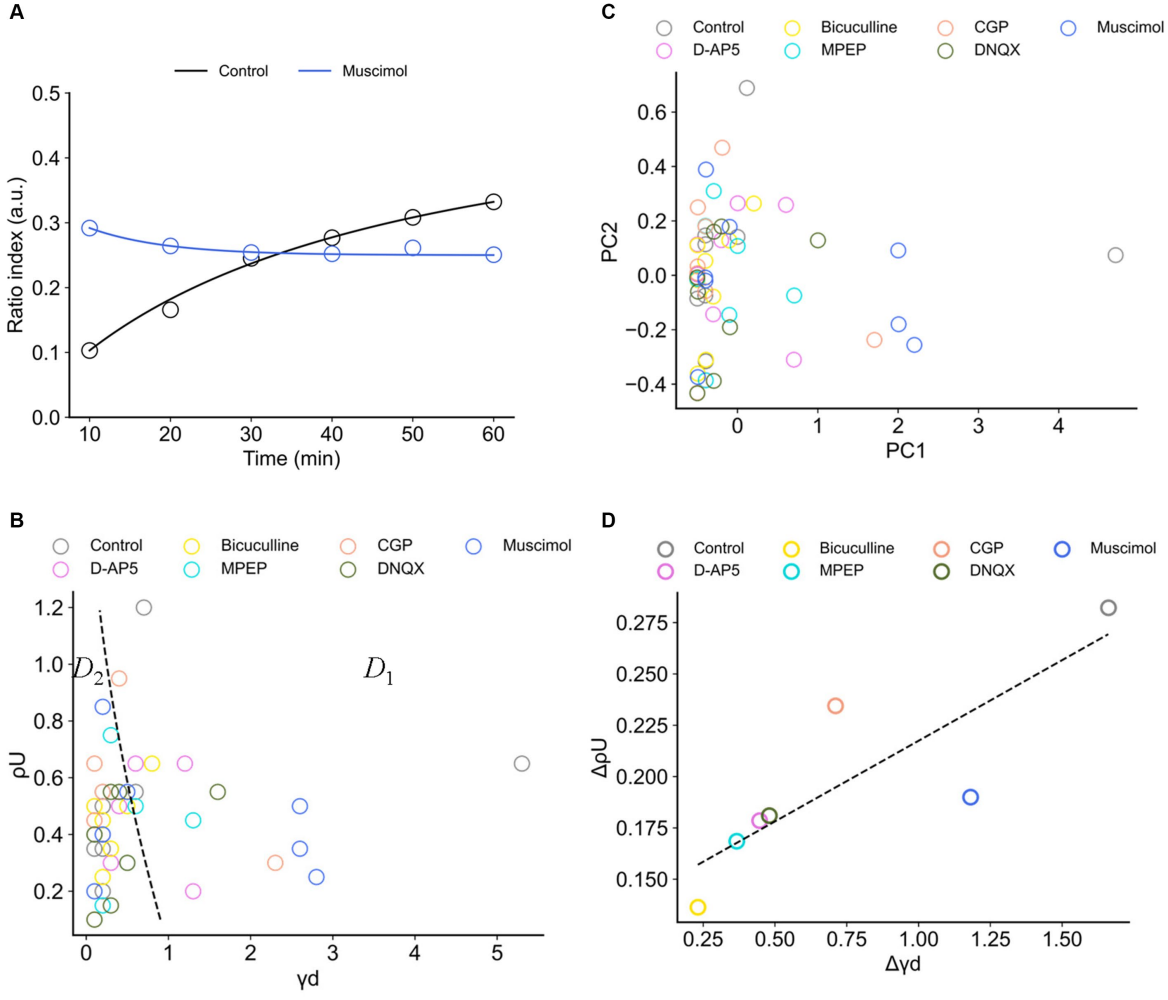
Synaptic efficacy models obtained from ratio indices (RIs) of experimental data sets. **(A)** Typical two examples for the fitting of RI data sets. The blue and black curves are the model trajectories obtained from muscimol and control conditions, respectively. Experimental data points are indicated by circles. **(B)** The distribution of two parameters, (i) the depression rates ($\gamma_{d}$) and (ii) the unstable fixed point ($\rho_{U}$), is illustrated. The dashed curve separates two regions *D*_1_ and *D*_2_. *D*_1_ (*D* < 0 in Equation 6) is the region where there exists only one stable fixed point, whereas *D*_2_ (*D* ≥ 0) is the region where there exist two stable fixed points. **(C)** The distribution of the data points in the first and second principal components (PC_1_ and PC_2_) of calculated coefficients in the model. **(D)** The scattering (variance) properties of $\gamma_{d}$ and $\rho_{U}$ (i.e., $△\;\gamma_{d}$ and $△\;\rho_{U}$, respectively) in the analyzed eight electrode sites for each drug condition are shown.

In all data sets under different drug conditions, the coefficients of the synaptic efficacy model were successfully acquired by minimizing EF (Equation 8) by searching for an optimal parameter set in a grid of the parameter space. The RI time series can be approximately represented by the simple model, and *ρ* trajectories of the model are in good agreement with the data points (Figure 9A). In addition, for example, Figure 9B shows the distribution of two parameters: (i) the depression rates ($\gamma_{d}$) and (ii) the unstable fixed point ($\rho_{U}$) separating the basins of attraction of the two stable states under all drug conditions. The other parameter sets are shown in Supplementary Figure 5.

In Figure 9B, the boundary of discriminant *D* (Equation 6) is also indicated by a black dashed line. In Figure 9B, the region indicated by *D*_1_ (*D* ≤ 0 in Equation 6) corresponds to one stable point, whereas the region indicated by *D*_2_ (*D* > 0) corresponds to two stable points. The obtained parameter sets ($\gamma_{d}$, $\rho_{U}$) are mostly distributed in Region *D*_2_, indicating the parameter sets are the coefficients of the model with a double-well potential. Among the drug conditions, the numbers of data points in the two regions are summarized in Table 3. As a result, there were more double-well potentials than single-well potentials in all conditions. We also calculated PCA distribution with estimated parameter sets in the first and second principal components (PC_1_ and PC_2_; Figure 9C). The distribution of model parameter sets in the two-dimensional PCA space illustrates an analogy to the distribution in ($\gamma_{d},\rho_{U}$) space (Figure 9B), suggesting the two parameters are crucial information among all parameters.

**Table 3:** Numbers of monostable or bistable potentials in the total eight electrode sites.

Condition	Label	Number of monostable potentials	Number of bistable potentials
Control	—	3	5
D-AP5	Glu (−)	3	5
DNQX	Glu (−)	1	7
MPEP	Glu (−)	2	6
Bicuculline	GABA (−)	1	7
CGP	GABA (−)	2	6
Muscimol	GABA (+)	3	5

Furthermore, to evaluate the spatial dependencies of these parameters, the scattering of $\gamma_{d}$ and $\rho_{U}$ (i.e., $△\;\gamma_{d}$ and $△\;\rho_{U}$, respectively) in the analyzed eight electrode sites was calculated. As a result, the parameters in the control condition show the highest variety in both $△\;\gamma_{d}$ and $△\;\rho_{U}$ (Figure 9D). There was also a significant positive correlation between $△\;\gamma_{d}$ and $△\;\rho_{U}$ (*r* = 0.845, *p* < 0.05). In other words, the control-conditioned group had intrinsically spatial characteristics in the depression rates and the separation of two stable states. Thus, all agonist/antagonists’ groups indicated decreases in both $△\;\gamma_{d}$ and $△\;\rho_{U}$. In particular, glutamatergic groups (i.e., D-AP5, MPEP, and DNQX) had similar dispersions (Figure 9D).

**Figure 10:**
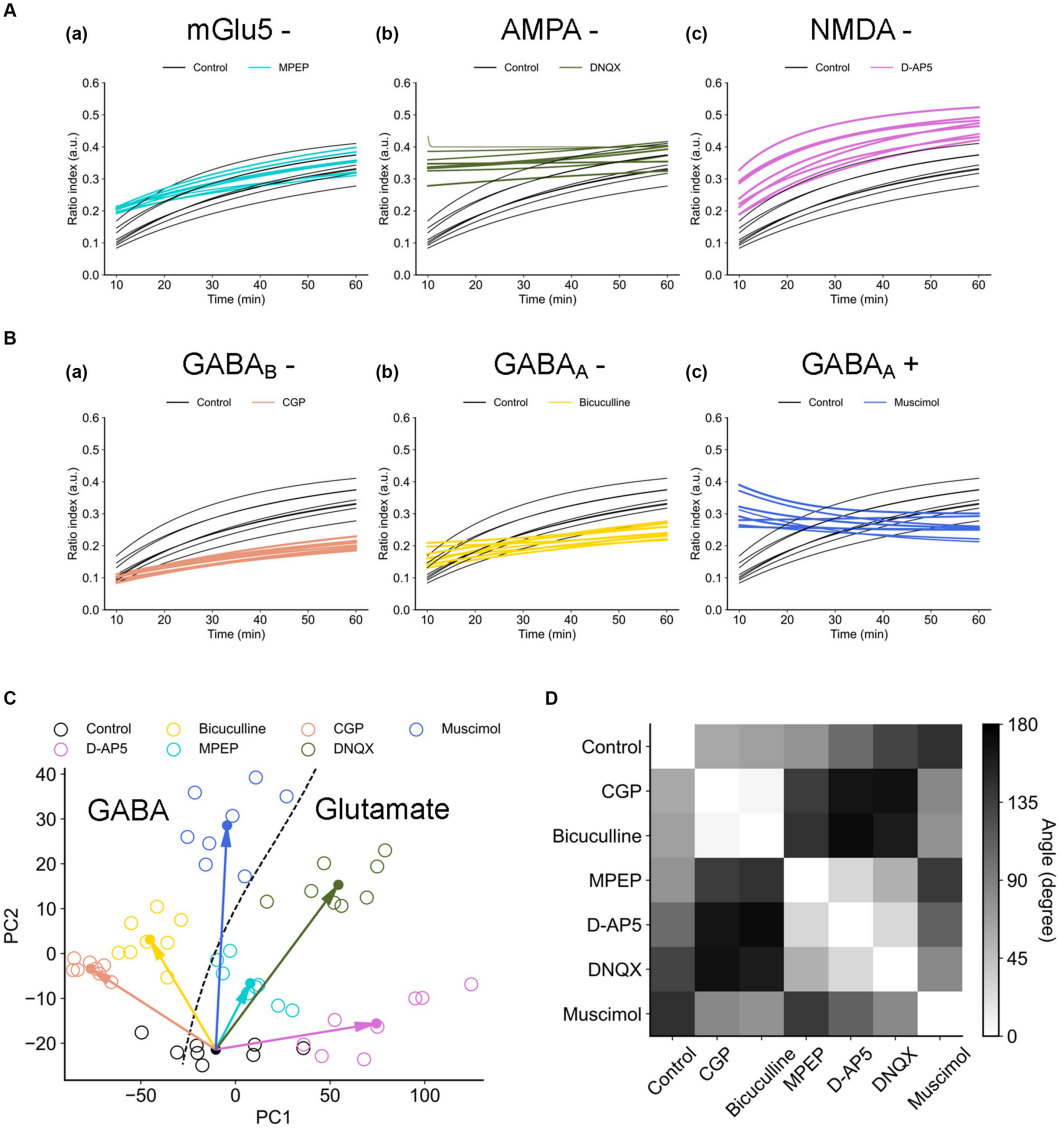
Trajectories of the synaptic efficacy models with the estimated coefficients under the different drug conditions. **(A)** Trajectories of the glutamate group. **(B)** Trajectories of the GABA group. **(C)** Distribution of data points on the first and second principal component space (PC_1_ vs. PC_2_). The dashed line indicates the boundary between two major groups (i.e., glutamate vs. GABA groups): the glutamatergic group (i.e., D-AP5, MPEP, and DNQX) and GABAergic group (i.e., bicuculline, CGP, and muscimol). **(D)** The angles of the paired centroid vectors among all groups are illustrated.

The transients of the synaptic efficacy model with the estimated coefficients are illustrated in Figures 10A,B. The temporal transients were specifically characterized among the condition groups. The transients of agonist/antagonist groups seemed to vary less compared to the control group in the time domain. To focus on two major groups (i.e., glutamate or GABA groups), the glutamatergic groups (i.e., D-AP5, MPEP, and DNQX) were on the right side of the boundary (Figure 10C). On the other hand, the GABAergic groups (i.e., bicuculline, CGP, and muscimol) were distributed on the left side of a classification boundary in two-dimensional PCA space (Figure 10C). As shown in two-dimensional PCA space, even for each condition group, they were clearly clustered.

In addition, to evaluate which receptor the most contributes to the formation of LTD dynamics in the mouse auditory cortex, each distance *d_i_* (*i* = 1, …, 6) between control and each agonist/antagonist group (Equation 9) was calculated (Figure 10C). As a result, the farthest group was D-AP5 (*d*_1_ = 85) and the others are as follows: DNQX (*d*_2_ = 74) > CGP (*d*_5_ = 69) > muscimol (*d*_6_ = 50) > bicuculline (*d*_4_ = 43) > MPEP (*d*_3_ = 23). The centroid vector of the muscimol group was directed with the largest angle to that of the control group with the largest angle, followed by DNQX and D-AP5 (see the left column of Figure 10D), and the glutamate group tended to have a different vector direction than the control.

**Figure 11:**
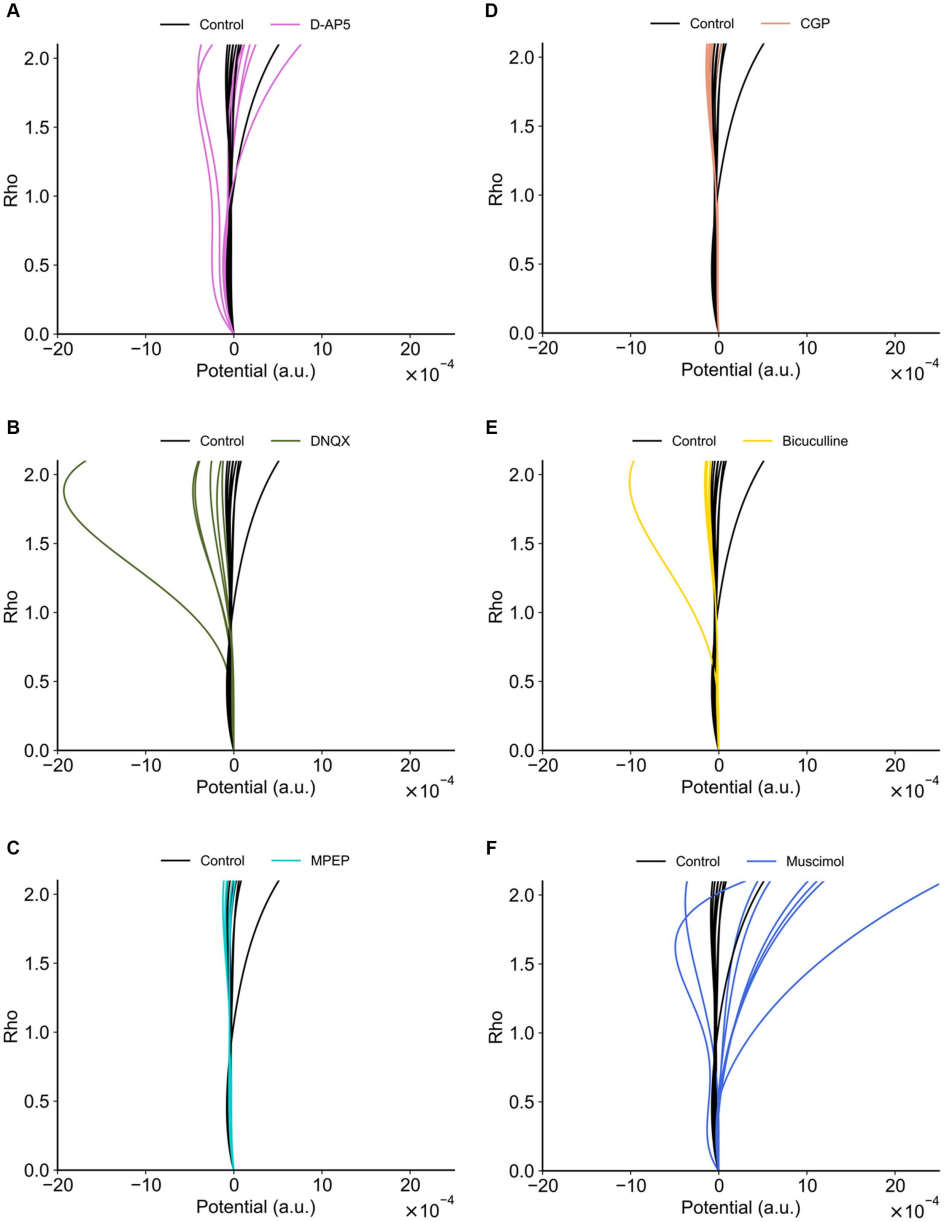
Potential functions concerning the synaptic efficacy models obtained from recorded RI data sets. **(A)** D-AP5 vs. control conditions. **(B)** DNQX. **(C)** MPEP. **(D)** CGP. **(E)** Bicuculline. **(F)** Muscimol. The potential functions of the control group are also superimposed on each plot. Furthermore, in each plot, eight potential functions were individually obtained from the eight nearest electrode sites around the TS site, suggesting the spatial variability among the recording sites (electrodes).

**Figure 12:**
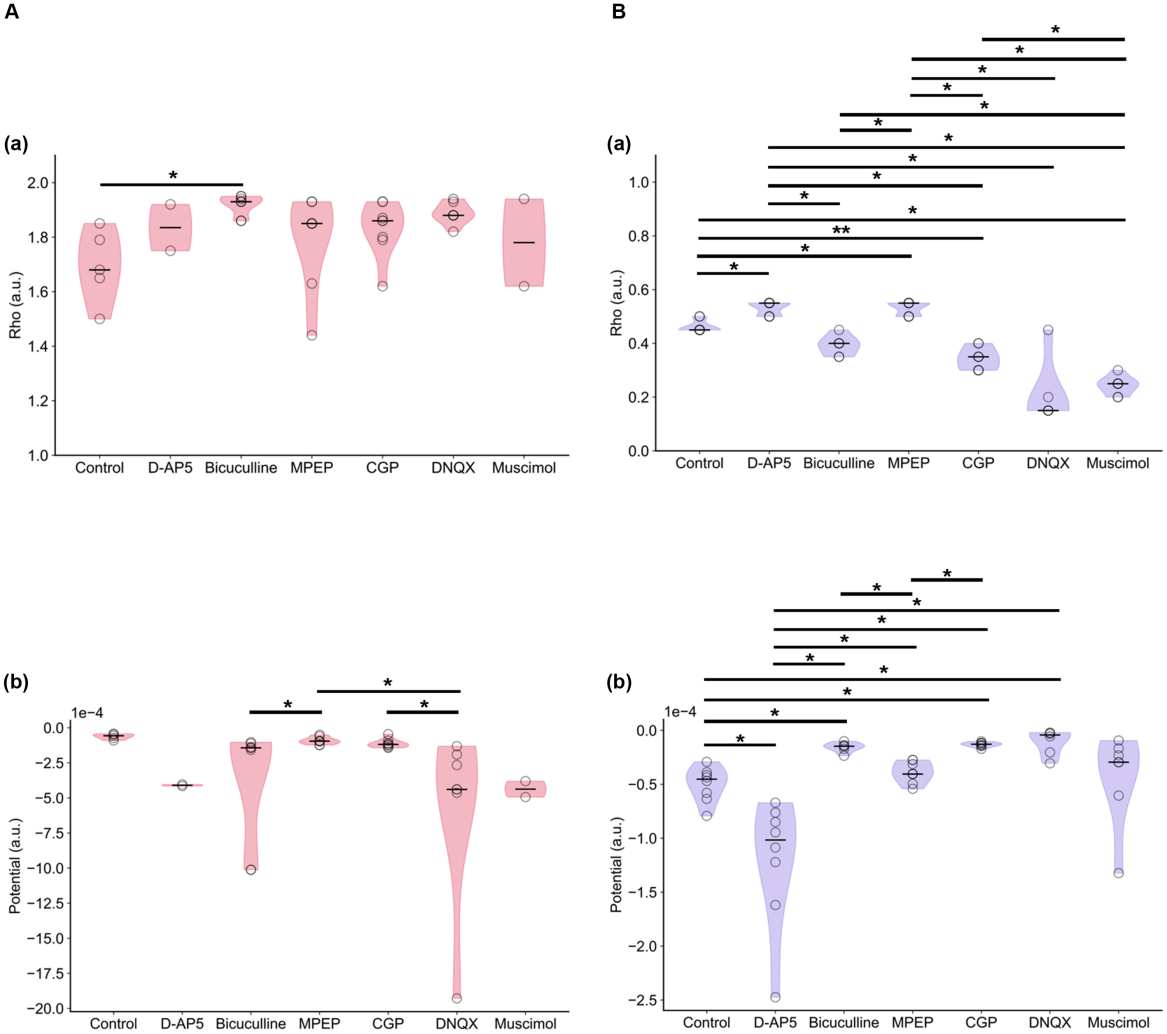
Stable fixed points and local minimal values of potential functions. **(A)** If two stable fixed points exist, the larger value of stable fixed points denoted by *ρ*_+_ corresponds to the LTP is illustrated. Stable fixed-point values (*ρ*_+_) in **(a)** and local minimal values [*V*(*ρ*_+_)] of potential functions (Equation 4) in **(b)** are illustrated under each drug condition. **(B)** Similarly, the stable fixed point denoted by *ρ* = *α* corresponds to the LTD is illustrated. Stable fixed-point values (*α*) in **(a)** and local minimal values [*V*(*α*)] of potential functions in **(b)** are illustrated under each drug condition. * and ** represent *p* < 0.05 and *p* < 0.01, respectively.

Figure 11 indicates the properties of potentials corresponding to synaptic model transients for recorded RI data sets. The potentials were estimated using Equation 4. Each group had both single- and double-well potential. The potentials of bicuculline and DNQX groups formed relatively deeper wells at higher *ρ*_+_ corresponding to the LTP state compared to the control group (Figure 12A). In contrast, the potentials of D-AP5 groups formed relatively deeper wells at *ρ* = *α* corresponding to the LTD state compared to the control group (Figure 12B), although most pairs concerning the control vs. each drug condition are significantly different in the potential depth values. The number of stable fixed points is summarized in Table 3.

## 4 Discussion

In this study, we characterized synaptic plasticity in auditory cortex slices by monitoring LFP responses at sites near (*d* ≤ 0.45 mm) and away (*d* > 0.45 mm) from a layer-4 TS site during electric short-pulse stimulation. We observed a marked depression of LFP responses immediately after local layer-4 TS within 0.45 mm from the TS site. To assess synaptic plasticity sensitivity to synaptic receptors, we administered agonist/antagonists for NMDA/non-NMDA, metabolic glutamate, and GABAergic receptors. Additionally, we constructed a simplified model using recorded response dynamics to elucidate the major receptors of TS-driven LTD in the mouse auditory cortex. Our approach captures evoked-response dynamics, including a transient state approaching a sustained state. Thus, we propose a method for LTD experiments to examine cortico-cortical synaptic plasticity in matured mouse auditory cortex (≥7 weeks) beyond the auditory critical period, with potential future applications in the medical treatment of hearing disorders.

### 4.1 LTD in brain slices, including the auditory cortex

With regard to neural plasticity in rodent brain slices, including the auditory cortex, several studies have reported that stimulation layer(s) and/or stimulation patterns can affect the type of long-term plasticity, i.e., LTP vs. LTD. Murakami et al. (2004) reported that after TS (100-Hz electrical pulses for 1 s) was applied to either of layers 2/3, 4, and 6 of rat brain slices, including auditory cortex, they found LTD or LTP occurred; response ratios (posterior vs. prior TS) recorded in layer 2/3 were 47 ± 6% (i.e., LTD for a TS site in layer 2/3), 125 ± 8% (LTP for TS in layer 4), and 170 ± 21% (LTP for TS in layer 6), respectively, compared with average peak LFP amplitudes before TS. Therefore, their result indicates that either LTP or LTD in rat brain slices exclusively occurs depending on the TS layer(s) in the cortex. In addition, Watanabe et al. (2007) reported that LTP and two types of LTD were present in brain slices obtained from the rat auditory cortex. They applied TS (100 Hz for 1 s) or low-frequency stimulation (LFS, 1 Hz for 900 s) to layer 5 and, respectively, found LTP and LTD from the field potential data recorded in layer 2/3. They also reported that LTD at a recording site in layer 2/3 was also induced by local TS applied to layer 2/3 0.3 mm away from the recording site. Their results also indicate that neither LTP nor LTD under the TS and LFS conditions was induced in the presence of an NMDA receptor antagonist, whereas an antagonist of metabotropic glutamate receptors had no effect on the LTP or LTD induction. In addition, LTDs induced by both LFS and local TS were suppressed in the presence of an antagonist of GABA_A_ receptors. Furthermore, to provide a more comprehensive understanding of the effects of stimulation protocols, Table 4 lists several examples from the literature regarding the impact of TS and other stimulation protocols on synaptic plasticity, both in brain slices *in vitro* and in various brain regions *in vivo*. These examples include validation experiments conducted in different cortical layers and brain regions, suggesting that the type of synaptic plasticity depends on the specific layer and/or brain region.

**Table 4:** Neural plasticity induced by stimulation patterns in brain areas.

Reference	Preparation	Targeted region	Stimulation pattern	Plasticity
Bliss and Lomo (1973)	Rabbit in vivo	Hippocampus	100 Hz for 3-4 s	LTP
Alger and Teyler (1976)	Rat slice	Hippocampus	0.1-15 Hz for 10 s	LTD
Dunwiddie and Lynch (1978)	Rat slice	Hippocampus	100 Hz for 1 s	LTP
Kimura et al. (1989)	Rat slice	Visual cortex, white matter	5 Hz for 30 – 60 s	LTP
Kudoh and Shibuki (1994)	Rat slice	Auditory cortex, white matter	100 Hz for 1 s	LTP
Tekök and Krnjević (1996)	Rat slice	Hippocampus	100 Hz for 1 s	LTP
Wanda et al. (2001)	Mouse slice	Hippocampus	100 Hz for 1 s	LTP
Shen et al. (2003)	Rat slice	Subthalamic nucleus	60 repetitive bursts consisting of 50 pulses delivered at 100 Hz	LTD
Murakami et al. (2004)	Rat slice	Auditory cortex, layer 2/3	100 Hz for 1 s	LTD
Murakami et al. (2004)	Rat slice	Auditory cortex, layers 4 & 6	100 Hz for 1 s	LTP
Watanabe et al. (2007)	Rat slice	Auditory cortex, layer 2/3	100 Hz for 1 s	LTD
Braz et al. (2007)	Mouse in vivo	Medial prefrontal cortex	100 Hz for 1 s	LTD

In this study, local TS was applied to layer 4, which is the recipient layer, from the projection of the auditory MGB, and field potentials were alternatively elicited by test stimuli applied at either TS or no-TS sites in layer 4. When the distance (*d*) between the TS site in layer 4 and a recording electrode site was within 0.45 mm (*d* ≤ 0.45 mm), a marked depression of field potentials was always observed immediately after local TS (Figure 3) in the laminar (vertical) and horizontal directions. Additionally, we found that LTD in all layers was induced under the conditions utilizing the extracellular perfusion of NMDA/non-NMDA receptor antagonists. Furthermore, the LTD difference between the control condition and the two experimental conditions utilizing the NMDA- and non-NMDA receptor antagonists was limited to a short time period (duration of 10–20 min) after TS in layer 4. Our results show that LTD induced by layer 4 TS was maintained over 40 min after the TS with a small amount of recovery (Figures 3C, 4), indicating that LTD in layer 4 was robust through cortico-cortical neural connections in the auditory cortex.

### 4.2 Neural circuits underlying LTD induced by local tetanic stimulation

Watanabe et al. (2007) reported that LTD of supragranular (layer 2/3) field potentials was induced by local TS (100 Hz for 1 s) applied to supragranular layers (layer 2/3) 0.3 mm from the recording site in the rat auditory cortex. Therefore, the local TS has been employed for the induction of LTD in cortical slices. Our protocol of neural plasticity induction using TS applied to layer 4 of mouse cortical slices is analogous to that used in Watanabe et al. (2007), although the targeted layers were different (layer 2/3 vs. layer 4). Watanabe et al. (2007) also reported that, after low-frequency stimulation (1 Hz for 900 s) was applied to layer 5, supragranular (layer 2/3) field potentials exhibited LTD. Their results indicate that the type of synaptic plasticity (LTP or LTD) is dependent on stimulation layers and/or patterns.

Although the cortico-cortical circuit underlying LTD is still unclear, we speculate the following possibility. In a neural circuit including the auditory cortex, when TS was applied to layer 4, synapses between layer 2/3 pyramidal neurons and interneurons were activated following the excitation of pyramidal neurons in layer 4. Then, the firing of postsynaptic neurons was inhibited through the action of local inhibitory interneurons, which were also driven by the axon collaterals of pyramidal neurons in layer 4. Therefore, the activities of postsynaptic terminals did not completely follow those of presynaptic terminals in the activated presynaptic neurons for a short-time window; postsynaptic depolarization during the activation of presynaptic terminals was small owing to the inhibition exerted by local interneurons. Rather, the activated timings of pre- and postsynaptic terminals were mostly reversed in a random fashion in cortico-cortical circuits, so that LTD was dominantly induced in the synapses between most pyramidal neurons in layer 4 according to the typical Hebbian rule or the spike-timing-dependent synaptic plasticity, i.e., presynaptic and postsynaptic activations are mostly in an inverse order.

Another possibility underlying the LTD induction is discussed in terms of postsynaptic calcium concentrations. To induce LTP/LTD of pyramidal cell connections, it is widely accepted that the key signal is postsynaptic calcium, which enters dendritic spines through NMDA receptors and/or voltage-dependent calcium channels (VDCCs; Chindemi et al., 2022). The postsynaptic calcium transients can subsequently trigger the expression of plasticity in either presynaptic and/or postsynaptic mechanisms. Therefore, the “calcium-control” hypothesis is one of the most common assumptions on how calcium dynamics are linked to changes in synaptic efficacy. In the calcium-control hypothesis, calcium transients of large amplitude are postulated to induce LTP; on the other hand, prolonged calcium transients of moderate amplitude would result in LTD. However, other forms considering multiple calcium sensors have also been proposed. *In vitro* experimental study reports on location-dependent synaptic plasticity indicate that connection-type specificity could be compatible with a uniform plasticity mechanism which is shaped by the physiological properties of the synaptic connection. Therefore, further experimental studies will be needed to understand the mechanisms underlying the LTD induction we observed in this report.

### 4.3 Physiological roles of LTD induced by local tetanic stimulation

Under physiological situations, high-frequency firing or bursting of pyramidal neurons in layer 4 of the auditory cortex can probably occur, for example, in response to the sound of amplitude and frequency modulation signals (Gaese and Ostwald, 1995; Liang et al., 2002). Repetitive neural activities driven by such modulation signals can induce LTD in the synapses between the active neuron and the surrounding pyramidal neurons in a cortico-cortical circuit because both the direct excitatory inputs and indirect inhibitory inputs to the surrounding pyramidal neurons are activated simultaneously in a nearly random fashion. As a result, this kind of LTD can possibly avoid the non-specific spreading of hyperexcitability to the surrounding neurons. This form of depression is referred to as “surround depression” (Watanabe et al., 2007), which might be induced by mechanisms similar to those underlying the LTD induced by the local TS in this study. In contrast, LTP in the synapses between the active neurons in the auditory cortex and neurons projecting from the MGB to the cortex will be induced in a finely tuned manner, where the neurons in both areas of the auditory pathway will be precisely synchronized within a small time window, leading to LTP through spike-timing-dependent plasticity (Zhu et al., 2013, 2014).

### 4.4 Experimental observations of LTD in long-term recordings of brain slices

In our physiological experiments during a long-term recording, one of the most difficulties is distinguishing LTD from the reduction of neural excitability in brain slices. More precisely, it is not easy to distinguish between the depressive responses that occurred in LFPs after TS and excitability reduction in brain slices for a long-term recording. The argument is that LTD in this study was induced only locally around TS. Thus, we tried to avoid the drawback by examining if the level of test stimulus-evoked neural activity at the recording sites away from a TS stimulation site remained unchanged during a series of experiments during the recording time (Supplementary Figure 2). Accordingly, the measuring system reported in this study has the advantage of using the MEA substrate because multisite recording and stimulation are easily realized in the system presented here. This measurement system and our experimental protocol will help to obtain more reliable electrophysiological data than a conventional method using single-site stimulation and recording experiments.

Here, we consider that LTD-like depression could be induced by any other factors at the neural level, such as (i) neural energy depletion, (ii) calcium overload, and/or (iii) neurotransmitter depletion. (i) First, the more activity of neurons increases, the more energy sources, including adenosine triphosphate (ATP) are required for cellular processes in the brain. In particular, excessive excitability, such as seizure activity, is known to induce neuronal energy depletion and consequent neural damage (Kovac et al., 2013). In glycolysis as a cellular process, ATPs are produced in the tricarboxylic acid (TCA) cycle from glucose molecules, which are included in the ACFS bath solution (11.0-mM D-glucose for this study). To rule out neuronal energy depletion as a cause of reduced response in each brain slice, we proposed a recording protocol while simultaneously monitoring the activity levels at many recording sites, including the sites more than 0.45 mm away from a TS site. Using the protocol, we have confirmed that the level of test stimulus-evoked activity at the recording sites away from the TS remains unchanged. Thus, our results indicated no significant changes in the indicator (i.e., responses away from the tetanic stimulation sites) following our stimulation protocol, suggesting that the observed LTD is not due to energy depletion. However, we cannot rule out the energy depletion in a very specific area around a TS electrode site on a brain slice, although diffusion in a slice chamber could provide glucose molecules in the bath solution to cells in a brain slice constantly. Therefore, further experiments observing the metabolic indicators, for example, levels of NAD(P)H and FAD (Palandt et al., 2024), are needed to investigate the neural energy depletion. (ii) Second, the main pathways of calcium ion influx from the extracellular matrix into cells are likely to be calcium-permeable ionotropic receptors and/or calcium ion channels. Neurons could be damaged and dead when calcium ions are overloaded through receptors and ion channels (Lee et al., 2016). In the neocortex, the primary excitatory neurotransmitter is glutamate. Glutamate binds to calcium-permeable ionotropic receptors that are also activated by NMDA or AMPA receptors. These NMDA and AMPA receptors are predominantly present in dendritic spines but also exist in peri-synaptic regions (Verma et al., 2018). If the primary cause of the observed LTD was calcium overload, the LTD would not have been observed in the application of antagonists of glutamatergic receptors. However, even in the low transparency of calcium ions, the LTD was observed (Figure 5), suggesting that the observed LTD was not mainly due to calcium overload or calcium toxicity in cells. (iii) Third, short-term synaptic depression during high-frequency stimulation may be explained by the depletion of presynaptic neurotransmitter vesicles, changes in vesicle release dynamics, and/or a reduction in presynaptic calcium (von Gersdorff and Borst, 2002). Regarding neurotransmitter depletion, paired-pulse afferent stimulation with a short inter-pulse interval is often used as a repetitive test-pulse stimulation to quantify the presynaptic release probability. However, because the repetitive paired-pulse stimulation protocol could alter the transient dynamics following TS, we did not apply such protocols in the current experiments. Therefore, we cannot rule out the possibility of LTD-like depression caused by a neurotransmitter depletion, and further experiments are planned to address this limitation in our future research.

### 4.5 Model analysis and its interpretation

Here, we constructed a simplified model and applied recorded response dynamics with the purpose of elucidating the major receptors of TS-driven LTD in the mouse auditory cortex. First, to apply the simplified model for evoked-response dynamics, we explored the optimized parameters of the dynamical model. Contracting the recorded response data into the coefficients of the differential equation (Equation 2) has the advantage to focus on the essential dynamics without being affected by unnecessary elements such as measurement noises and individual differences of responses in specific cortical recording sites. Second, we projected all estimated dynamics to the two-dimensional PCA space, and all groups were clustered. Our clustering method revealed the characteristic differences among the drug conditions using the an agonist and antagonists of synaptic receptors. In addition, under the drug conditions, the glutamate group and the GABA group were successfully separated in the trajectory space of the model (Figure 10C). This result suggests that all analyzed receptors have specific roles in the temporal dynamics to induce LTD in the mouse auditory cortex. Moreover, the cluster of D-AP5 was the farthest from the cluster of the control (Figure 10C), which suggests that NMDA receptors are possibly the largest contributor of LTD in the mouse auditory cortex in our analytical sense. To the best of our knowledge, no studies have used a synaptic model to reveal that NMDA receptors have an important role in TS-induced LTD as well as other receptors. In our future study, we would like to find any other significant receptors which could fill a blank space between the D-AP5 and DNQX groups in the two-dimensional PCA space (Figure 10C). Because this blank space is classified and occupied as the glutamate receptor region, unknown glutamatergic receptors we did not examine in this study could possibly appear.

Next, we analyzed the state transition with regard to the potentials (*V* in Equation 4) and the corresponding discriminant (*D* in Equation 6). First, the potentials had a deeper well at the higher equilibrium point corresponding to LTP in DNQX, bicuculline, and CGP compared to the control. In contrast, the number of equilibrium points did not seem to be different among the drug groups. Thus, it is noteworthy that AMPA, GABA_A_, and GABA_B_ receptors had individual roles to form potentials to pull up the LTD state. Furthermore, GABA_A_ and GABA_B_ receptors are known to have inhibitory effects. In our findings, the GABAergic inhibitory role seemed to be functional in the LTD potential region. In addition, it is known that cerebellar long-term depression (LTD) is characterized by a reduction in synaptic AMPA receptor expression (Huganir and Nicoll, 2013). To the best of our knowledge, this study represents the first instance of suggesting that synapses blocked by AMPA receptors exhibit a greater potential for LTP than LTD, on the basis of the modeling analysis using empirical data from the electrophysiological experiments.

Furthermore, we contracted experimental data sets into the parameter (i.e., *α*, *γ*_*d*_, *ρ_U_*, and *ρ*_*0*_) space of the model equation of Equation 2 and compared it with the PCA space, in which the contribution rates of the first two components are sufficiently high (>98%). Subsequently, we compared the parameter set distribution in more detail, using the two different reductional spaces with two dimensions: the original parameter space vs. the PCA space. We found that the depression rate (*γ*_*d*_) and the unstable fixed point (*ρ*_*U*_) separating the basins of attraction of the two stable states (i.e., *ρ* = *α* and *ρ* = *ρ*_+_ if two stable fixed points exist) were significant in the characterization of LTD dynamics because the parameter distribution in the two-dimensional PCA space is profoundly similar to that of the *γ*_*d*_–*ρ*_*U*_ space (Figure 9B vs. Figure 9C). These two parameters (*γ*_*d*_ and *ρ*_*U*_) also showed the highest spatial dependency in the control, although lower in other agonist/antagonist groups (Figure 9D). In Equation 2, *γ*_*d*_ is included in the cubic function in the first term on the right-hand side, which mainly determines the faster dynamics of the temporal evolution. In contrast, *ρ*_*U*_ is included in the additional linear term on the right-hand side, which determines the slower dynamics of the variable to the sustained state. Therefore, our result obtained by the analysis is quite reasonable for understanding and interpreting the model dynamics in terms of the time scale.

### 4.6 Significance and conclusion of this study

Examining the synaptic plasticity of cortico-cortical connections in the matured mouse auditory cortex (≥7 weeks) beyond the auditory critical period will be beneficial because we expect a future application of brain stimulation in the medical treatment of hearing disorders and artificial auditory neuroprostheses. Furthermore, a combination of finer multisite extracellular recordings with patch-clamp recordings at a cellular level will help to improve the understanding of the underlying LTD mechanisms in our future study.

In conclusion, our study sheds new light on neural plasticity induced by tetanic stimulation in the mouse auditory cortex *in vitro*. We observed a marked depression of field potential responses after local layer-4 tetanic stimulation within 0.45 mm from the site. LTD in layer 4 persisted against NMDA/non-NMDA receptor antagonists. We developed a simplified synaptic efficacy model, elucidating the major receptors involved in TS-driven LTD. Our analysis reveals the contribution and significance of all the analyzed receptors for LTD. This suggests intrinsic spatial properties of TS-induced LTD maintained by various neurotransmitter receptors. Future studies are needed to understand neuromodulation in hearing loss and adaptive changes in auditory cortex plasticity in clinical and rodent models.

## Data availability statement

The raw data supporting the conclusions of this article will be made available by the authors, without undue reservation.

## Ethics statement

The animal study was approved by Institutional Animal Care and Use Committee of Hokkaido University. The study was conducted in accordance with the local legislation and institutional requirements.

## Author contributions

RF: Conceptualization, Data curation, Formal analysis, Funding acquisition, Investigation, Methodology, Project administration, Resources, Software, Supervision, Validation, Visualization, Writing – original draft, Writing – review & editing. KK: Data curation, Formal analysis, Investigation, Software, Writing – original draft. TT: Conceptualization, Data curation, Formal analysis, Funding acquisition, Investigation, Methodology, Project administration, Resources, Software, Supervision, Validation, Visualization, Writing – original draft, Writing – review & editing.
