# Supplementary material for: Analyzing the transient response dynamics of long-term depression in the mouse auditory cortex in vitro through multielectrode-array-based spatiotemporal recordings
Source: Front Neurosci. 2024 Sep 12;18:1448365. doi: 10.3389/fnins.2024.1448365 (PMC11424455; doi:10.3389/fnins.2024.1448365)
Supplement: Supplementary file 1 [file Data_Sheet_1.PDF]

Supplementary materials

Analyzing the Transient Response Dynamics of Long-term Depression in the Mouse Auditory Cortex *in vitro* through Multielectrode-array-based Spatiotemporal Recordings

Ryo FURUKAWA[1], Kouta KUME[1], Takashi TATENO[2*]

[1]Graduate School of Information Science and Technology, Hokkaido University, Kita 14, Nishi 9, Kita-ku, Sapporo, 060-0814 Japan

[2]Faculty of Information Science and Technology, Hokkaido University, Kita 14, Nishi 9, Kita-ku, Sapporo, 060-0814 Japan

* Correspondence:
Professor Takashi TATENO
tateno@ist.hokudai.ac.jp

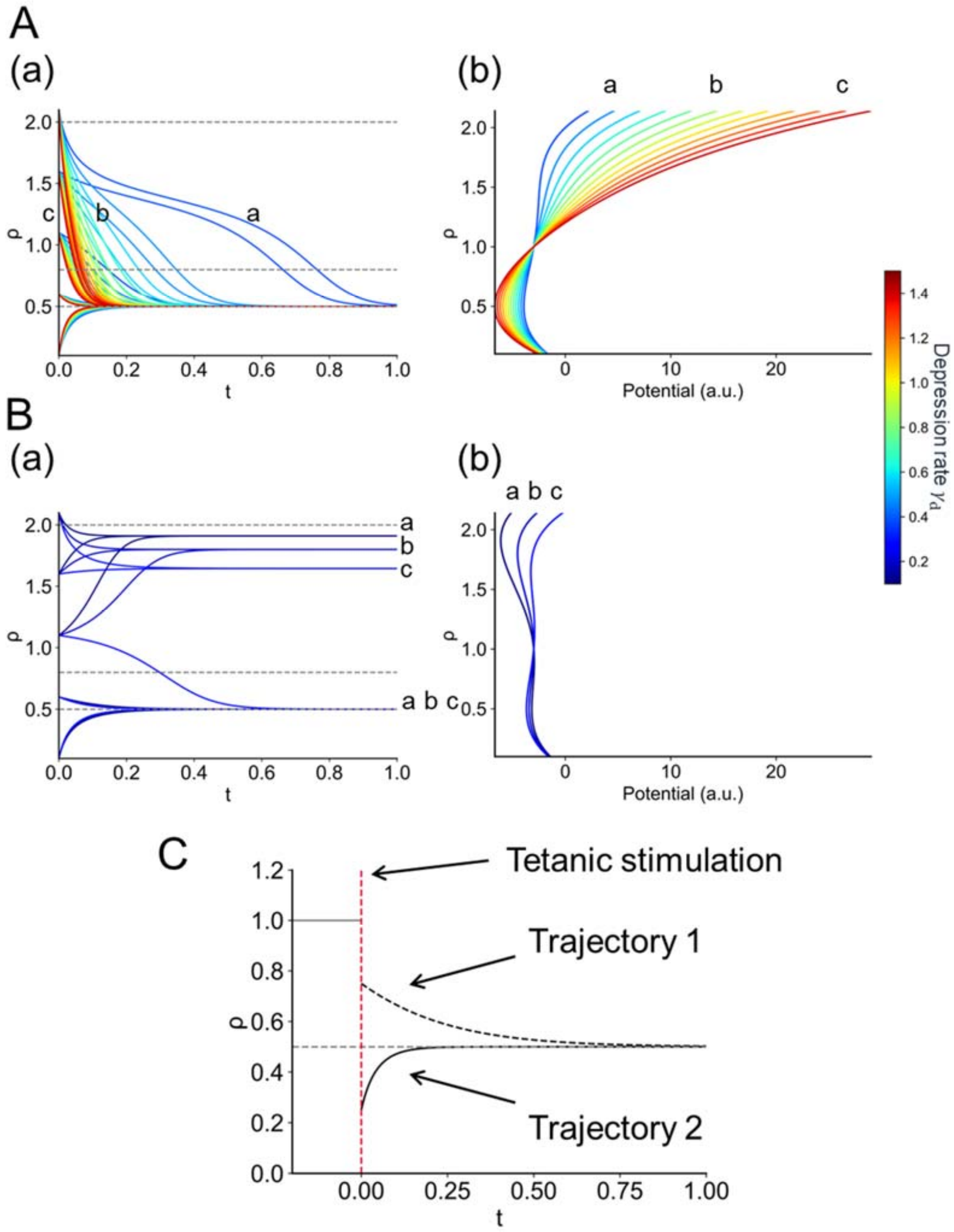

Supplementary Fig. 1. **The trajectories of the synaptic efficacy model and its potential functions.** (A) In the model, the state variable ρ describes the dynamics of the synaptic efficacy, its trajectories $\rho(t)$ in (a) and potential functions $V(\rho)$ in (b) are illustrated for several initial conditions; model parameters are $\rho_0 = 0.1$ to 2.1 with 0.5 step, $\alpha = 0.5$, $\rho_U = 0.8$, $\gamma_d = 0.1$ to 0.3 with 0.1 step, and $\tau = 0.05$. (B) Similarly, the trajectories $\rho(t)$ in (a) and potential functions in (b). The model parameters are $\rho_0 =$ 0.1 to 2.1 with 0.5 step, $\alpha = 0.5$, $\rho_U = 0.8$, $\gamma_d = 0.4$ to 1.5 with 0.1 step, and $\tau = 0.05$. (C) Two examples of the model trajectories. The synaptic efficacy variable ρ of the original Graupner and Brunel model is shifted upward to $+\alpha$. For easy understanding of LTD without normalization before TS, the lower stable point ($\rho = \alpha$) concerning the depression is set to be 1.0 (i.e., $\alpha = 1.0$) in our model.

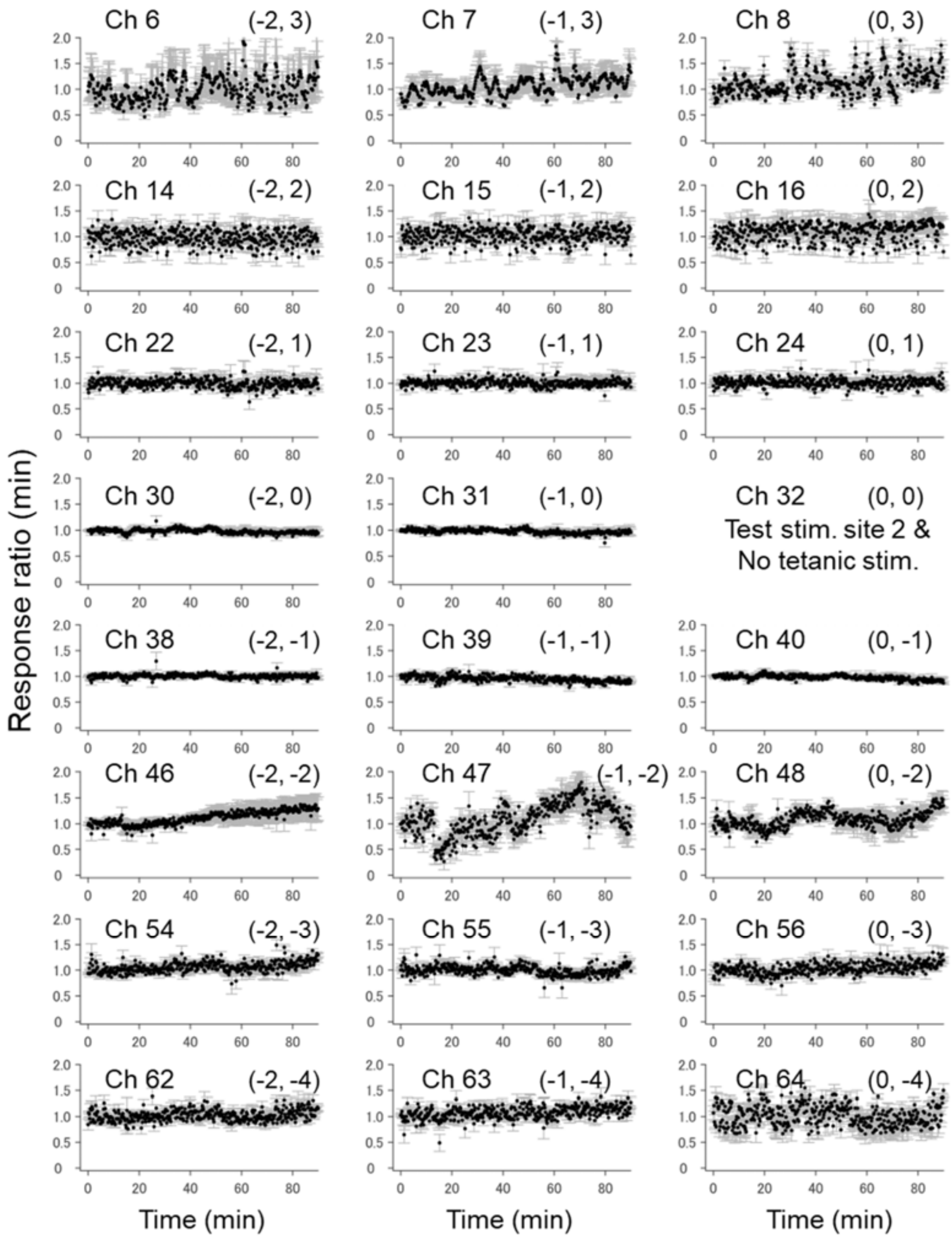

Supplementary Fig. 2. The time courses of LTD around the non-TS and test stimulation site in the cortical laminar layers prior and posterior to TS. The average time courses of LTD for 23 electrode sites at three columns are illustrated on average for five brain slices (n = 5). The non-tetanic and test stimulation site is represented as ch 32, which was the reference point (the origin (0,0)) in a two-dimensional matrix. To express each element in the 2D matrix, a bracket representation (*a*, *b*) denotes an element in the two-dimensional matrix, where *a* and *b* respectively represent the row and column number relative to the origin (0, 0). For example, the coordinate representation (−1, 2) indicates that the position (ch 15) relative to the test stimulation electrode (ch 32) is one inter-electrode distance (150 μm) on the left horizontally and the inter-electrode distance of two intervals (300 μm) vertically above ch 32.

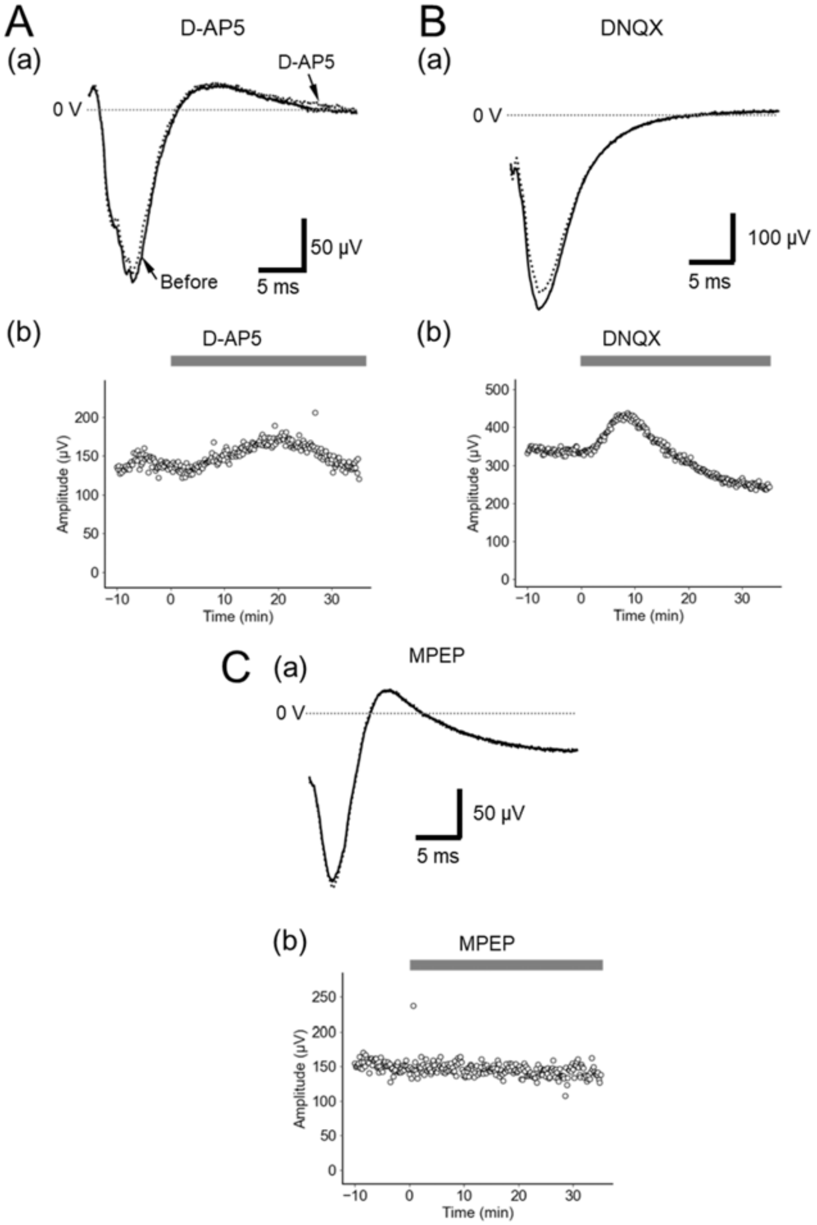

Supplementary Fig. 3. (A) LFP responses before and after NMDA receptor antagonist (10 µM D-AP5) application. Typical response waveforms before and 30 min after D-AP5 administration are shown in (a). Time course of negative-going peak amplitudes is illustrated during the whole recording period (50 min) in (b). D-AP5 was applied at time 0 min in the plot. (B) LFP responses before and after non-NMDA receptor antagonist (50 µM DNQX) application. Typical response waveforms before and 30 min after DNQX administration are shown in (a). Time course of negative-going peak amplitudes is illustrated during the whole recording period (45 min) in (b). TS was applied at time 0 min in the plot. (C) LFP responses before and after a metabolic glutamate receptor antagonist (10 µM MPEP) application. Typical response waveforms before and 30 min after MPEP administration are shown in (a). Time course of negative-going peak amplitudes is illustrated during the whole recording period (45 min) in (b). MPEP was applied at time 0 min in the plot.

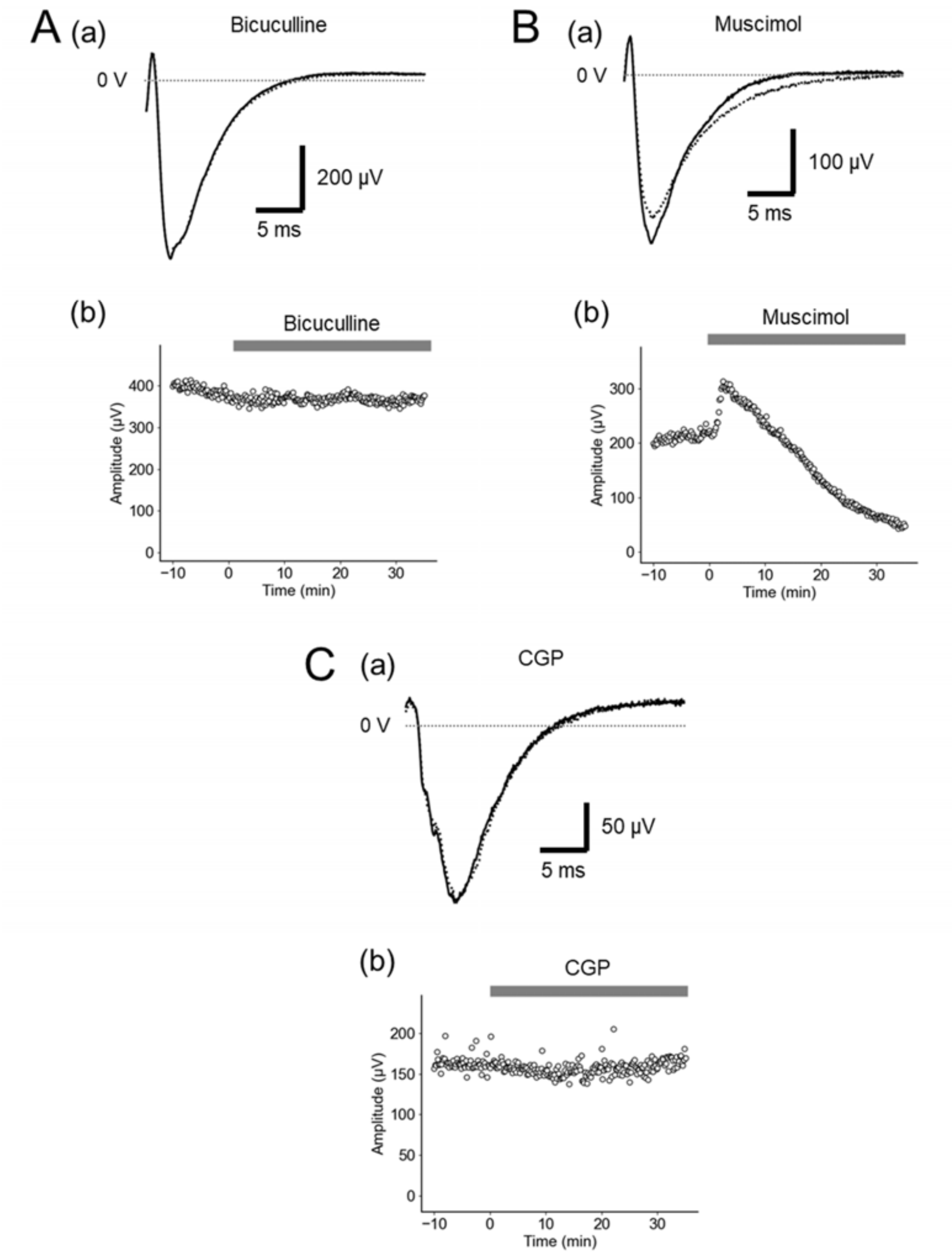

Supplementary Fig. 4. (A) LFP responses before and after $GABA_A$ receptor antagonist (5 µM bicuculline) application. Typical response waveforms before and 30 min after bicuculline administration are shown in (a). Time course of negative-going peak amplitudes is illustrated during the whole recording period (45 min) in (b). Bicuculline was applied at time 0 min in the plot. (B) LFP responses before and after NMDA receptor antagonist (5 µM muscimol) application. Typical response waveforms before and 30 min after muscimol administration are shown in (a). Time course of negative-going peak amplitudes is illustrated during the whole recording period (45 min) in (b). Muscimol was applied at time 0 min in the plot. (C) LFP responses before and after $GABA_B$ receptor antagonist (5 µM CGP) application. Typical response waveforms before and 30 min after CGP administration are shown in (a). Time course of negative-going peak amplitudes is illustrated during the whole recording period (45 min) in (b). CGP was applied at time 0 min in the plot.

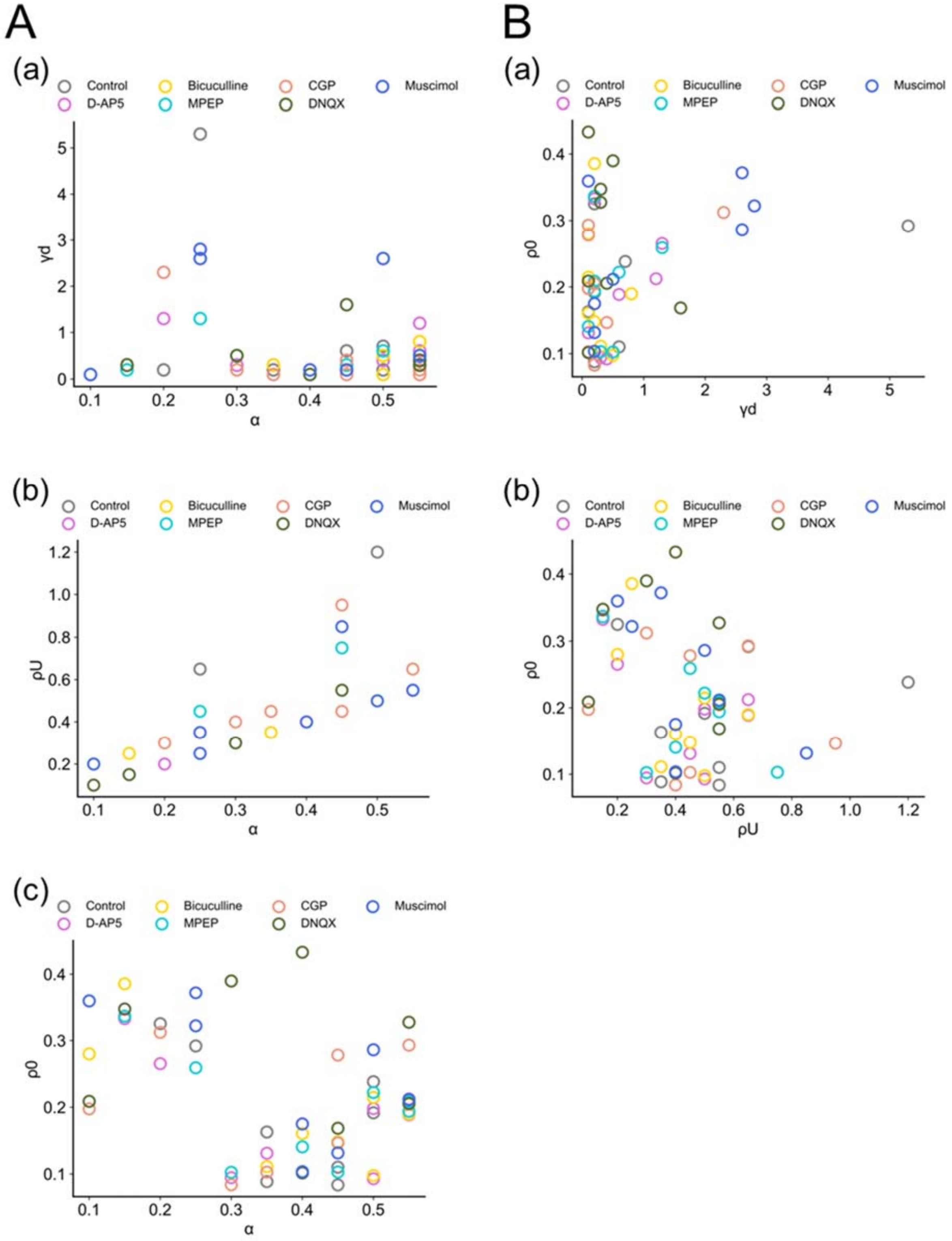

Supplementary Fig. 5. Estimated parameter points on two-dimensional parameter spaces. (A) In (a), (b), and (c), the distribution of parameter points on the two-dimensional spaces (α vs. γ_d), (α vs. ρ_U), and (α vs. ρ_0) is respectively illustrated. (B) In (a) and (b), similarly, the two-dimensional spaces are respectively (ρ_0 vs. γ_d) and (ρ_0 vs. ρ_U).
